# The miR-124-3p/Neuropilin-1 Axis Contributes to the Proliferation and Metastasis of Triple-Negative Breast Cancer Cells and Co-Activates the TGF-β Pathway

**DOI:** 10.3389/fonc.2021.654672

**Published:** 2021-04-12

**Authors:** Jiayang Zhang, Xuesong Zhang, Ziyi Li, Qingshan Wang, Yan Shi, Xian Jiang, Xueying Sun

**Affiliations:** ^1^ Key Laboratory of Carcinogenesis and Translational Research (Ministry of Education/Beijing), Department of Breast Oncology, Peking University Cancer Hospital & Institute, Beijing, China; ^2^ Department of General Surgery, Heilongjiang Provincial Hospital, Harbin Institute of Technology, Harbin, China; ^3^ The Hepatosplenic Surgery Center, The First Affiliated Hospital of Harbin Medical University, Harbin, China; ^4^ Department of Pathology, The Second Affiliated Hospital of Harbin Medical University, Harbin, China

**Keywords:** neuropilin-1, triple-negative breast cancer, microRNA-124-3p, epithelial-mesenchymal transition, transforming growth factor-β, proliferation, metastasis

## Abstract

Triple-negative breast cancer (TNBC) accounts for 90% of breast cancer-associated mortality. Neuropilin-1 (NRP-1) acts as a non-tyrosine kinase receptor for several cellular signaling pathways involved in the proliferation and metastasis of cancer cells. However, the miRNAs that regulate NRP-1 expression and the underlying mechanisms in TNBC cells remain unclear. In the present study, we found that TNBC cells expressed higher levels of NRP-1 than non-TNBC cells. Stable transfectants depleted of NRP-1 were generated from two TNBC cell lines, human MDA-MB-231 and mouse 4T1 cells. NRP-1 depletion significantly suppressed the proliferation of TNBC cells by arresting the cell cycle at phase G0/G1 by upregulating p27 and downregulating cyclin E and cyclin-dependent kinase 2. NRP-1 depletion also repressed cell migration and epithelial-mesenchymal transition (EMT) by inducing the upregulation of E-cadherin and the downregulation of N-cadherin, matrix metalloproteinase (MMP)-2 and MMP-9, and reducing MMP-2 and MMP-9 activities as detected by gelatin zymography assay. By applying multiple miRNA-target prediction tools, we screened potential miRNAs with binding sites with the 3’-untranslated region of the NRP-1 gene and selected 12 miRNA candidates, among which miR-124-3p displayed the most vigorous activity to downregulate NRP-1 as validated by luciferase assay and miRNA transfection assay. By downregulating NRP-1, miR-124-3p mimics inhibited the proliferation, migration, and invasion of TNBC cells, and antagomiR-124-3p could partially abolish the effects of NRP-1 depletion. In the animal experiments, NRP-1 depletion inhibited tumorigenesis and liver metastasis of TNBC cells, while miR-124-3p mimics inhibited the growth of established TNBC tumors. In the mechanistic exploration, we revealed that NRP-1 co-interacted with transforming growth factor (TGF)-β to activate the TGF-β pathway, which regulates EMT-related molecules. In summary, the present results indicate that the miR-124-3p/NRP-1 axis contributes to the proliferation and metastasis of TNBC cells and co-activates the TGF-β pathway, suggesting that these molecules may present as potential therapeutic targets and valuable biomarkers for TNBC.

## Introduction

Breast cancer is the second most common cause of cancer-related deaths and remains a massive health burden for females worldwide (1). In particular, a subtype of breast cancer, triple-negative breast cancer (TNBC), lacks the expression of estrogen receptor, progesterone receptor, and human epidermal growth factor receptor-2 (HER-2) and accounts for 15% of all breast cancer cases (2). It is characterized by earlier recurrence, the tendency of metastasis, shortage of effective therapeutic drugs, and poor prognosis (2, 3). Despite the successful development of therapeutics for other subtypes of breast cancer, challenges remain in the management of TNBC. Therefore, it is needed to explore the molecular mechanisms accounting for the progression of TNBC.

Neuropilin-1 (NRP-1) acts as a non-tyrosine kinase receptor to exert functions in cancer by co-activating vascular endothelial growth factor (VEGF), hepatic growth factor (HGF), epidermal growth factor (EGF), and transforming growth factor (TGF)-β signaling pathways (4–8), which are decisive in the progression of TNBC (9–12). NRP-1 is shown to contribute to the metastasis of breast cancer (13–15), and its expression level correlates inversely with the survival of breast cancer patients (16).

MicroRNAs (miRNAs) are a type of non-coding RNAs containing 19-25 nucleotides and regulate over 60% of genes involved in a wide range of biological processes (17). Increasing evidence indicates that miRNAs are associated with the progression and metastasis of breast cancer (18, 19). For instance, miR-10b is highly expressed in metastatic breast cancer cells and positively regulates cell migration and invasion by downregulating homeobox D10 (20). Several miRNAs have been demonstrated to regulate NRP-1 expression and participate in the progression of various types of cancer (7, 21–23). However, it’s unknown what upstream mRNAs regulate NRP-1 expression in breast cancer, especially TNBC. We, therefore, designed this study to seek potential miRNAs and investigate the underlying mechanisms contributing to the proliferation and metastasis of TNBC cells.

## Materials and Methods

### Cell Culture, Antibodies and Reagents

Human luminal A type of breast cancer MCF-7 and ZR-75-1 cells, human TNBC MDA-MB-231 and MDA-MB-453 cells, human mammary epithelial cells (HMEpC), and mouse TNBC 4T1 cells of BALB/cfC3H strain were purchased from the cell bank of Chinese Academy of Sciences (Shanghai, China). MCF-7 and HMEpC cells were cultured in Dulbecco’s Modified Eagle Medium (DMEM) (Gibco BRL, Grand Island, NY, USA), while MDA-MB-231, MDA-MB-453, and ZR-75-1 cells were cultured in DMEM/Nutrient Mixture F-12 medium (Thermo Fisher Scientific, Shanghai, China). 4T1 cells were cultured in RPMI-1640 medium (Thermo Fisher Scientific, Shanghai, China), supplemented with 10% FBS (fetal bovine serum) in a humidified atmosphere of 5% CO_2_. The cell lines had been authenticated to be negative for mycoplasma infection with a short tandem repeat analysis using a PCR-based Universal Mycoplasma Detection kit (American Type Culture Collection, Manassas, VA, USA). The detailed information for antibodies, reagents, and kits is shown in **Table S1** in **Supplementary Material**.

### Establishment of Stable Transfectants Depleted of NRP-1

The methods have been previously described (6, 24). In brief, an NRP-1 shRNA pSuppressorNeo vector targeting NRP-1 gene sequence (GGACAGAGACTGCAAGTAT) (corresponding to nucleotides 519-537 of human NRP-1 [RefSeq transcript: NM_003873.7] and nucleotides 721-739 of mouse NRP [RefSeq transcript: NM_008737.2]) and a scrambled shRNA vector (Sc-shRNA) were constructed. MDA-MB-231 and 4T1 cells were seeded in 10-cm dishes, grown to approximately 67% confluence, and then transfected with 4 μg of each vector by using Lipofectamine2000. Cells were detached by trypsinization 48 h after transfection and seeded in the selection medium containing geneticin (500 μg/ml). Stable transfectants were selected after 3-4 weeks of culture, and from MDA-MB-231 and 4T1 cells, four stably transfected cell lines, namely MDA-MB-231-NRP^low^, MDA-MB-231-Sc, 4T1-NRP^low^, and 4T1-Sc cells were generated, respectively.

### Luciferase Assay and Plasmid Constructs

The full-length of the 3’-untranslated region (UTR) of human NRP1 mRNA (NCBI Gene ID: 8829) was cloned into a pMIR-REPORT luciferase reporter vector (Ambion) to generate an SV40 promoter-driving luciferase reporter vector, which was used as a wild-type vector for miR-124-3p because it contained the putative miR-124-3p binding sequence (CUAUGUCCUCUCAAGUGCCUUUUUG). This binding sequence was mutated to “CUAUGUCCUCUCAACACGGAAUUUG” to generate a mutated vector. Luciferase reporter transfection and assay were performed as described previously (7). Briefly, the above vectors and an empty vector without 3’-UTR of NRP-1 were transfected into cells. Luciferase activities in cells were measured by using a luciferase assay kit (Promega, Madison, WI), and miRNA function was expressed as the percentage of the luciferase activity of the reporter vector with 3’-UTR of NRP-1 over that of the empty vector.

### Animal Experiments

Three sets of animal experiments were carried out to examine the role of NRP-1 and/or miR-124-3p on the growth of TNBC tumors and liver metastasis. Female 6-8-week nude BALB/c-nu/nu mice and BALB/c mice were purchased from the SLAC Laboratory Animal Co., Ltd. (Shanghai, China). Animals were housed at the Animal Research Center, the First Affiliated Hospital of Harbin Medical University. The protocols have been approved by the Institute Animal Ethics Committee with a permit (No. SYXK20020009), which complies with the Experimental Animal Regulations by the National Science and Technology Commission, China.

#### Tumorigenesis Study

Two groups (n=6) of six BALB/c-nu/nu mice received a subcutaneous injection of MDA-MB-231-Sc or MDA-MB-231-NRP^low^ cells (5 × 10^6^) into the flank, respectively. Animals and their palpable tumors were monitored for 4 weeks, and then animals were euthanized and tumors harvested, weighed, imaged, and analyzed.

#### Therapeutic Effects of miR-124-3p

MDA-MB-231 cells (5 × 10^6^) were injected subcutaneously into the flank of BALB/c-nu/nu mice. Around 10 days later, palpable tumors reached ~100 mm^3^ in volume and mice were randomly assigned to 3 groups (n=8), in which intratumoral injections of vehicle, negative control oligonucleotides, or miR-124-3p mimics were given, respectively. The vehicle was prepared by mixing an equal volume of serum-free medium and Lipofectamine2000 and was used to prepare transfection solution with oligonucleotides. Each tumor received a 50µl of injection solution containing 200µg of oligonucleotides. Three days after injection, 2 mice in each group were sacrificed and tumors were removed for detecting gene expression. The remaining tumors received injections on days 5, 10, and 15, and were measured every four days. The mice were monitored and euthanized on day 20.

#### Liver Metastasis Study

4T1-Sc or 4T1-NRP^low^ cells (4 × 10^4^ suspended in 50 µl of PBS) were injected into the left inguinal mammary fat pad of BALB/c mice, which were randomly divided into two groups (n=8). Mice were monitored for 35 days and then euthanized. The liver was harvested and fixed with 4% paraformaldehyde and transverse sections were prepared at 5 different levels to cover the entire liver. The sections were stained with hematoxylin and eosin (HE), metastatic nodules containing more than 6 tumor cells were counted, and the mean number of nodules was recorded as the number of metastases.

### Other Analyses and Assays

All the other analyses and assays used in the study have been reported previously (6, 23, 25, 26), and are described in detail in the **Supplementary Material**.

### Statistical Analysis

GraphPad Prism 8.02 (GraphPad Software, San Diego, CA, USA) was employed for statistical analyses. Data are expressed as mean values ± standard deviation. Multiple comparisons were made with a one-way analysis of variance (ANOVA) followed by a Tukey post-hoc test. Comparisons between two groups were made by a t-test. The relationship between the expression of NRP-1 and miR-124-3p was analyzed by using Pearson’s correlation coefficient. P < 0.05 was considered statistically significant.

## Results

### TNBC Cells Expressing Higher Levels of NRP-1 and the Validation of Stably Transfected Cells

**Figure 1:**
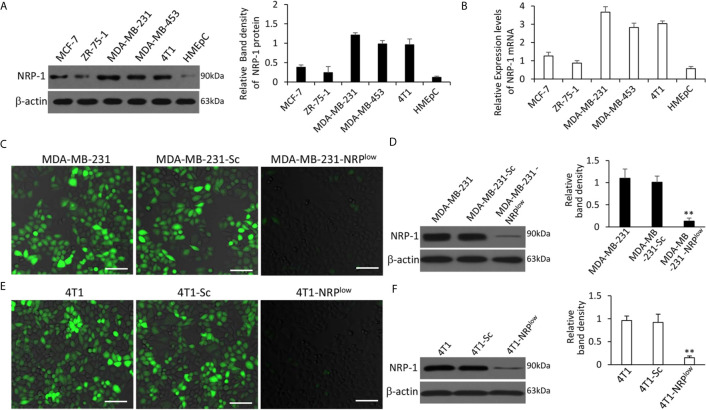
Expression of NRP-1 in breast cancer cells and validation of stable transfectants. **(A, B)** A panel of cell lines were lysed and subjected to Western blot analysis for detecting expression of NRP-1 protein **(A)** and qRT-PCR for measuring the expression of NRP-1 mRNA **(B)**. **(C–F)** MDA-MB-231 and 4T1 cells were genetically modified to generate stably transfected MDA-MB-231-Sc, MDA-MB-231-NRP^low^, 4T1-Sc and 4T1-NRP^low^ cells, respectively. NRP-1 expression (stained green) in the above cells was examined by fluorescent immunocytochemistry **(C, E)** and Western blot analysis **(D, F)** with an anti-NRP-1 Ab. Scale bar, 50 μm. Band density was normalized to β-actin. “** P<0.001” (one-way ANOVA with a Tukey post-hoc test) indicates a significant downregulation from corresponding parental cells.

The order of cell lines with the highest to the lowest expression of NRP-1 was MDA-MB-231, MDA-MB-453, 4T1, MCF-7, ZR-75-1 and HMEpC cells; and TNBC cells (MDA-MB-231, MDA-MB-453 and 4T1) expressed higher levels of NRP-1 than non-TNBC cells (MCF-7 and ZR-75-1) (**Figures 1A, B**). Compared with parental cells, MDA-MB-231-NRP^low^ cells expressed significantly lower, while MDA-MB-231-Sc cells expressed similar levels of NRP-1 and were used as controls (**Figures 1C, D**). Similarly, 4T1-NRP^low^ cells expressed significantly lower, while 4T1-Sc cells expressed similar levels of NRP-1, compared with 4T1 cells (**Figures 1E, F**).

### NRP-1 Promotes the Proliferation of TNBC Cells

**Figure 2:**
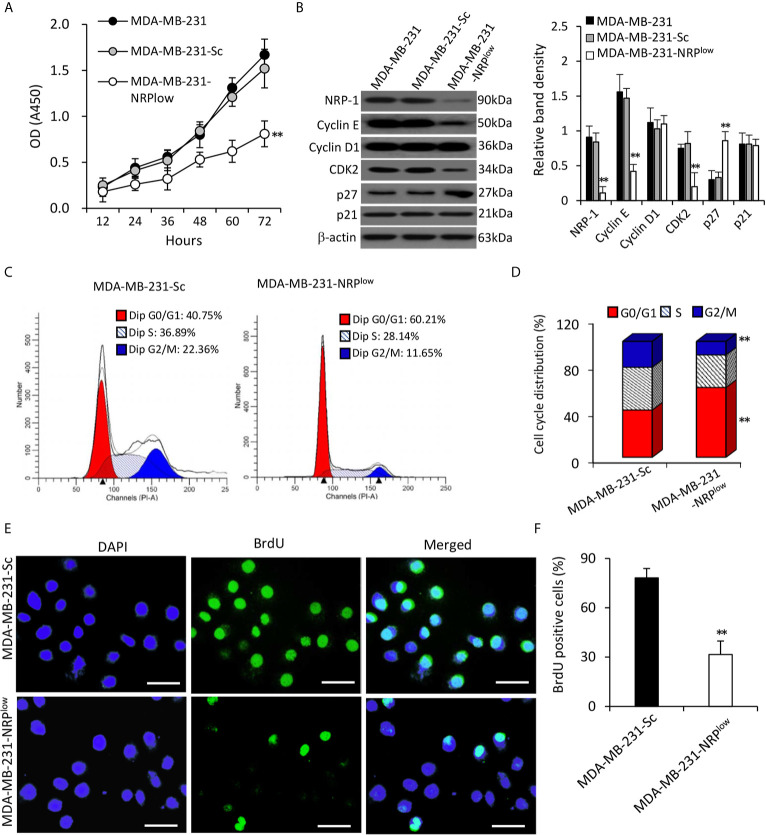
Depletion of NRP-1 inhibits the proliferation of MDA-MB-231 cells. **(A)** MDA-MB-231, MDA-MB-231-Sc and MDA-MB-231-NRP^low^ cells were cultured and the optical density (OD) was determined at indicated time points. **(B)** Cells were subjected to Western blot analysis for detecting the expression of proliferation-related proteins. Band density was normalized to β-actin. **(C–F)** MDA-MB-231-Sc and MDA-MB-231-NRP^low^ cells were cultured for 48 h. **(C, D)** Cell cycle distribution was detected by flow cytometry **(C)** and percentages of cells at different phases were charted **(D)**. **(E, F)** BrdU incorporation assay was employed to detect cell proliferation. Representative images were taken from 4’,6-diamidino-2-phenylindole (DAPI)-stained nuclei of all cells (blue, left column), BrdU-stained nuclei of proliferating cells (green, middle column), and merged photographs (right column). Magnification bar = 20 µm. **(F)** The percentage of BrdU-positive cells was plotted. “**P < 0.001” [one-way ANOVA with a Tukey post-hoc test in **(A, B)**, t-test in **(D, F)**] indicates a significant difference from MDA-MB-231-Sc cells.

MDA-MB-231-NRP^low^ cells had significantly lower viability, but MDA-MB-231-Sc cells had similar viability, compared with MDA-MB-231 cells (**Figure 2A**). The depletion of NRP-1 significantly downregulated the expression of cyclin E and cyclin-dependent kinase 2 (CDK2), but not had little effect on cyclin D1, in agreement with the previous studies (6, 7). For further exploring the upstream factors that regulate cyclin E and CDK2, we found that NRP-1 depletion significantly upregulated the expression of p27 but not p21 (**Figure 2B**). Since cyclin E and CDK2 are key regulators for cell cycle, we examined cell cycle distribution by using flow cytometry, which showed that 60.21% of MDA-MB-231-NRP^low^ cells were arrested at the G0/G1 phase, which was significantly higher than that in MDA-MB-231-Sc cells (40.73%) (**Figures 2C, D**). The BrdU incorporation assay showed that depletion of NRP-1 significantly inhibited cell proliferation as 31.6% of MDA-MB-231-NRP^low^ cells were BrdU-positive, which was significantly lower than that of MDA-MB-231-Sc cells (78.2%) (**Figures 2E, F**).

### Seeking and Identifying Potential miRNAs that Regulate NRP-1 in TNBC Cells

**Figure 3:**
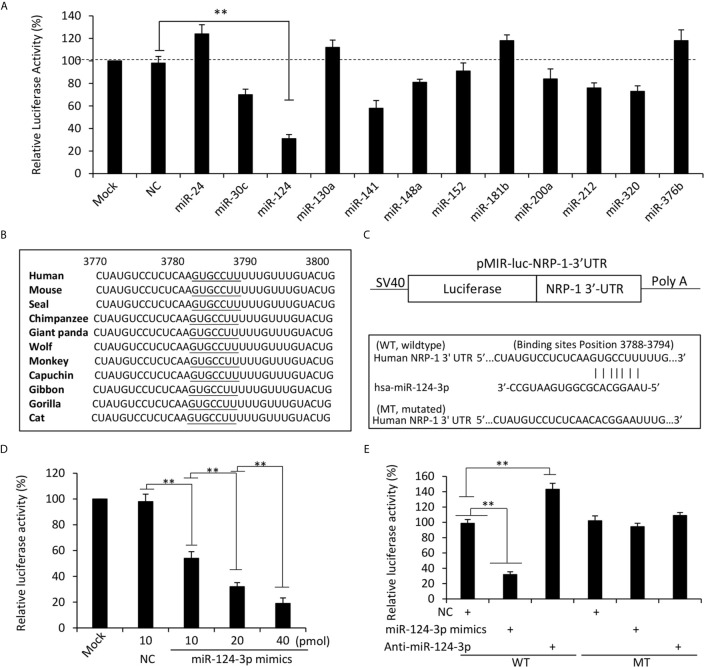
MiR-124-3p regulates NRP-1 expression by binding to the 3’UTR. **(A)** MDA-MB-231 cells were co-transfected with a luciferase reporter containing sequences of the 3’UTR of human NRP-1 gene with negative control (NC) oligonucleotides or each of the miRNA mimics. Mock-transfected cells served as controls. **(B)** A highly conserved miR-124-3p binding site on the 3’UTR of NRP1 is observed in all the species. **(C)** Two luciferase reporter vectors containing the wild-type (WT) or mutated (MT) NRP-1 3’-UTR were constructed. **(D)** MDA-MB-231 cells were co-transfected with the WT luciferase reporter and miR-124-3p mimics at different concentrations. **(E)** MDA-MB-231 cells were co-transfected with WT or MT luciferase reporters, and NC, miR-124-3p mimics or antagomiR-124-3p oligonucleotides. Luciferase activities were measured and normalized to untreated cells as a control. “**P <0.001” (one-way ANOVA with a Tukey post-hoc test).

We next screened potential miRNAs that have putative binding sites with the 3’UTR of human NRP-1 gene by using multiple miRNA prediction tools including miRanda (https://omictools.com/miranda-tool), TargetScan (http://www.targetscan.org/), miRWalk (http://mirwalk.umm.uni-heidelberg.de/), mirdb (http://mirdb.org/), and miRTarBase (http://mirtarbase.mbc.nctu.edu.tw/), which identified 12 miRNA candidates (**Figure S1** in **Supplementary Material**). The full-length of 3’UTR of human NRP1 mRNA (NCBI Gene ID: 8829) was inserted into an SV40 promoter-driving luciferase reporter vector, which was co-transfected into MDA-MB-231 cells with each miRNA mimics or negative control oligonucleotides (**Table S2** in **Supplementary Material**). Among the 12 miRNAs, miR-124-3p displayed the strongest ability to inhibit the luciferase activity (**Figure 3A**). A further investigation showed a highly conserved miR-124-3p binding site on the 3’UTR of NRP1 in all the available species (**Figure 3B**) and thus miR-124-3p was selected for further investigation. A mutated luciferase reporter vector was constructed by inserting a mutated 3’-UTR of NRP-1 gene (**Figure 3C**). The inhibitory effect of miR-124-3p on the luciferase activity was shown to be in a dose-dependent manner (**Figure 3D**). MiR-124-3p mimics or antagomiR-124-3p significantly altered the luciferase activity in cells co-transfected with the wild-type vector but not the mutated vector (**Figure 3E**). The results indicate that the binding site on NRP-13’-UTR is essential for miR-124-3p to display its regulatory effect in MDA-MB-231 cells.

### NRP-1 Is Negatively Regulated by miR-124-3p in TNBC Cells

**Figure 4:**
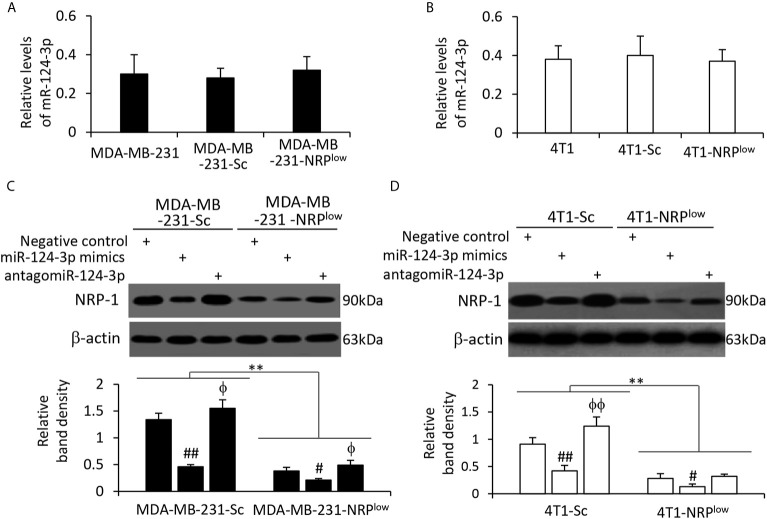
The alteration of NRP-1 expression by transfection of oligonucleotides targeting miR-124-3p. **(A, B)** MDA-MB-231, MDA-MB-231-Sc, MDA-MB-231-NRP^low^, 4T1, 4T1-Sc and 4T1-NRP^low^ cells were subjected to qRT-PCR to measure the expression level of miR-124-3p. **(C, D)** Cells as indicated were transfected with negative control, miR-124-3p or antagomiR-124-3p oligonucleotides for 48 h and then subjected to Western blotting analysis. Band density was normalized to β-actin. A one-way ANOVA with a Tukey post-hoc test was used for statistical analysis. “**P < 0.001” indicates a significant difference; “^ϕ^P < 0.05” and “^ϕϕ^P < 0.001” indicate a significant increase, while “^#^P < 0.05” and “^##^P < 0.001”, a significant reduction, compared with negative control-transfected cells.

NRP-1 depletion had shown no effect on miR-124-3p expression in MDA-MB-231 cells (**Figure 4A**) and mouse 4T1 cells (**Figure 4B**). Human miR-124-3p (MIMAT0000422) and mouse miR-124-3p (MIMAT0000134) share the same gene sequence (http://www.mirbase.org). MiR-124-3p mimics transfection resulted in the downregulation of NRP-1, while antagomiR-124-3p transfection led to an increase in NRP-1 expression, compared with negative control, in both human MDA-MB-231 cells (**Figure 4C**) and mouse 4T1 cells (**Figure 4D**). The results are in agreement with a highly conserved miR-124-3p binding site on the 3’UTR of NRP1 in all the species (**Figure 3B**).

By applying qRT-PCR analyses, we detected the expression of miR-124-3p in available cell lines and found that MDA-MB-231 cells expressed the lowest level of miR-124-3p (**Figure S2A** in **Supplementary Material**). By using a Pearson test, we analyzed the correlation of miR-124-3p expression levels with the expression levels of NRP-1, and a negative correlation was observed (**Figures S2B, C** in **Supplementary Material**).

### MiR-124-3p Inhibits the Proliferation of TNBC Cells

**Figure 5:**
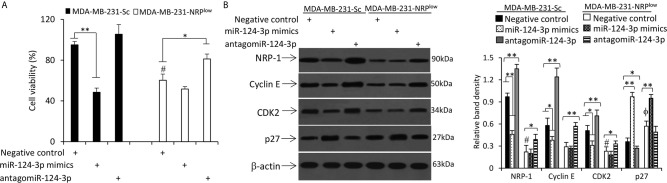
MiR-124-3p inhibits cell proliferation by downregulating NRP-1. MDA-MB-231-Sc and MDA-MB-231-NRP^low^ cells were transfected with negative control, miR-124-3p mimics or antagomiR-124-3p oligonucleotides and continued to be cultured for 48 h. **(A)** The viability of cells was detected by CCK-8 assay and normalized to mock-treated MDA-MB-231-Sc cells. **(B)** Cells were lysed and subjected to Western blot analysis. Band density was normalized to β-actin. “*” (P<0.05) and “**” (P < 0.001) indicate a significant difference. A one-way ANOVA with a Tukey post-hoc test was used for statistical analysis. “^ϕ^P < 0.05” indicates a significant increase, while “^#^P < 0.05”, a significant reduction, compared with negative control-treated MDA-MB-231-Sc cells.

Transfection of miR-124-3p mimics inhibited the proliferation of MDA-MB-231-Sc cells, while antagomiR-124-3p could partially abolish the reduced proliferation of MDA-MB-231-NRP^low^ cells by NRP-1 depletion (**Figure 5A** and **Figure S3** in **Supplementary Material**). Similar results were obtained in mouse 4T1 cells (**Figure S4** in **Supplementary Material**). In mechanistic exploration, the role of miR-124-3p on cell proliferation was supported by its regulatory effects on the expression of CDK2, cyclin E and p27 through downregulating NRP-1 expression (**Figure 5B**). Specifically, miR-124-3p mimics downregulated the expression of cyclin E and CDK2, and upregulated the expression of p27, while antagomiR-124-3p showed an opposite effect (**Figure 5B**). Since antagomiR-124-3p inhibits the negative regulatory function of miR-124-3p, which binds to the 3’-UTR of NRP-1 mRNA, while NRP-1 shRNA targets the coding sequence (CDS) of NRP-1 mRNA, two regulatory mechanisms on NRP-1 expression exist in MDA-MB-231-NRP^low^ cells transfected with antagomiR-124-3p. In addition, NRP-1 shRNA and antagomiR-124-3p may also transfect different cells because they are unable to transfect 100% of cells. Therefore, antagomiR-124-3p only partially abolished the effects of NRP-1 depletion (**Figure 5B**).

### MiR-124-3p Inhibits the Migration and Epithelial-Mesenchymal Transition of TNBC Cells by Downregulating NRP-1

**Figure 6:**
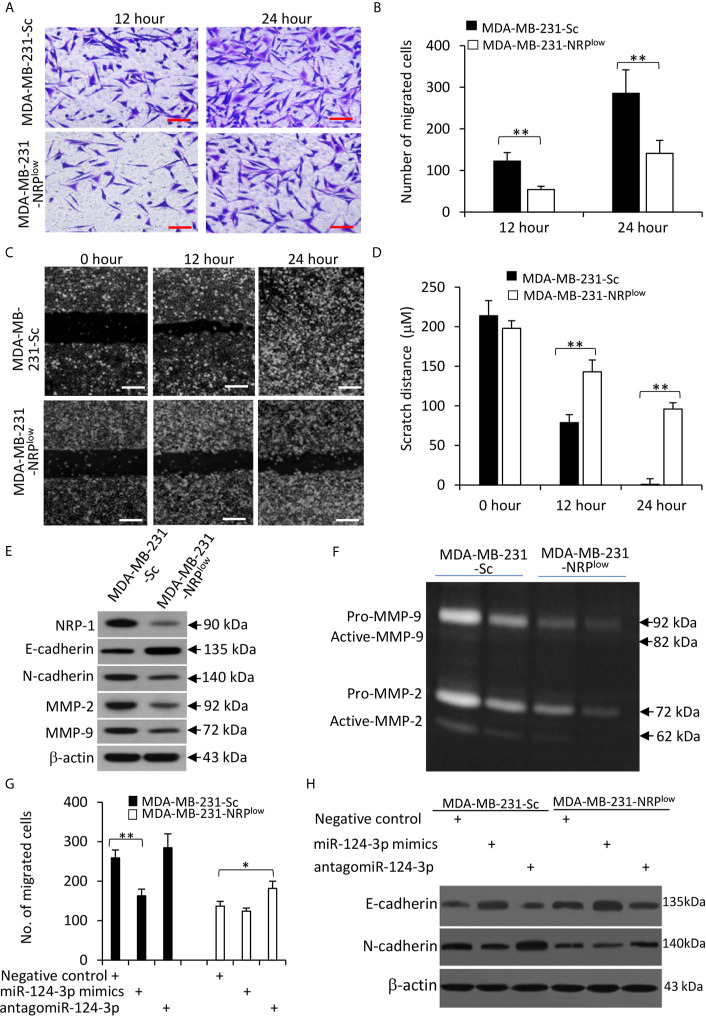
The miR-124-3p/NRP-1 axis regulates cell migration and EMT. **(A, B)** Transwell migration assays were used to evaluate the migrating ability of MDA-MB-231-Sc and MDA-MB-231-NRP^low^. **(A)** Migrated cells were stained with crystalline violet at 12 and 24 h. Magnification bar = 100 µm. **(B)** The numbers of migrated cells were counted. **(C, D)** The above cells were subjected to scratch migration assays. Scratch areas were imaged **(C)** and scratch distances were measured **(D)** at 0, 12 and 24 h. Magnification bar = 200 µm. **(E)** The above cells were subjected to Western blot analysis. **(F)** Conditioned media from MDA-MB-231-Sc and MDA-MB-231-NRP^low^ (2× 10^4^ or 1 × 10^4^) cells, which had been incubated for 48 h. Gelatin zymography assay was used to detect the activity of MMP-9 and MMP-2. **(G, H)** Cells were transfected with negative control, miR-124-3p mimics or antagomiR-124-3p oligonucleotides for 24 and then subjected to Transwell assays as in **(A)**. **(G)** Migrated cells were counted 24 h later. **(H)** Cells were subjected to Western blot analysis. “*P < 0.05” and “** P < 0.001” [t-test in **(B, D)**; one-way ANOVA with a Tukey post-hoc test in **(H)**] indicate a significant difference. “**P < 0.001” indicates a significant difference from MDA-MB-231-Sc cells.

NRP-1 has been shown to play a role in promoting the migration of cells from other cancer types (7, 23). Here we confirmed that NRP-1 depletion significantly inhibited the migrating ability of MDA-MB-231 cells by Transwell (**Figures 6A, B**) and scratch (**Figures 6C, D**) assays. It is well known that breast cancer cells gain their ability to migrate and invade through the epithelial-to-mesenchymal transition (EMT) (27). We showed here that NRP-1 depletion led to significantly downregulation of N-cadherin, matrix metalloproteinase (MMP)-2 and MMP-9, and upregulation of E-cadherin (**Figure 6E**). The results of gelatin zymography assays also showed that MDA-MB-231-NRP^low^ cells had lower activities of MMP-2 and MMP-9 (**Figure 6F**).

Furthermore, miR-124-3p mimics reduced the number of migrated cells, while antagomiR-124-3p partially abolished the effect of NRP-1 depletion on cell migration (**Figure 6G**); and similar results were obtained with mouse 4T1 cells (**Figure S4** in **Supplementary Material**). The above effects of miR-124-3p were in accordance with the changes in E-cadherin and N-cadherin expression (**Figure 6H**) and the results of Matrigel invasion assay (**Figure S5** in **Supplementary Material**).

### NRP-1 Depletion Suppresses the Growth and Liver Metastasis of TNBC Cells *In Vivo*

**Figure 7:**
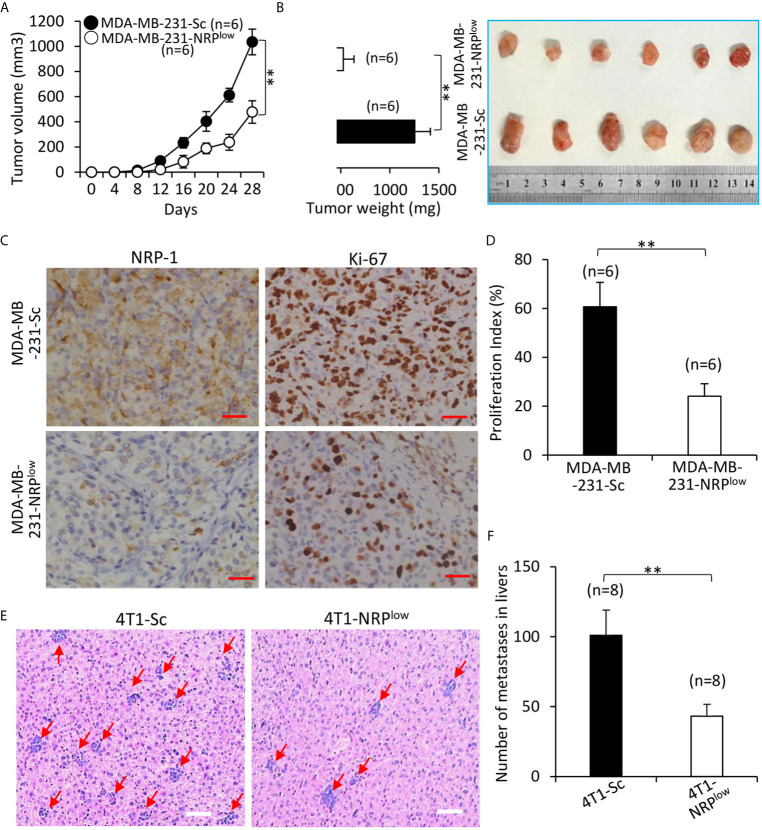
Depletion of NRP-1 inhibits the growth and liver metastasis of TNBC tumors in mice. **(A, B)** MDA-MB-231-Sc and MDA-MB-231-NRP^low^ cells were subcutaneously inoculated into two groups of BALB/c-nu/nu mice. **(A)** The size of tumors was measured at indicated time points. **(B)** Tumors were resected from mice, weighed and photographed 28 days after cell inoculation. **(C)** Illustrated are representative images from tumor sections, which were immunostained with anti-NRP-1 and anti-Ki-67 Abs. Magnification bar = 100 µm. **(D)** The cell proliferation index was quantified. **(E, F)** 4T1-Sc and 4T1-NRP^low^ cells were injected into the left inguinal mammary fat pad of BALB/c mice. Livers were taken 35 days later, stained with HE, examined under microscopy **(E)** and numbers of metastatic nodules were counted **(F)**. Magnification bar = 500 µm. Metastatic nodules in livers are pointed by arrows. “n” indicates the number of mice in each group. “**P < 0.001” (t-test).

Compared with control MDA-MB-231-Sc tumors, MDA-MB-231-NRP^low^ tumors grew significantly faster (**Figure 7A**), and were significantly larger (1035.4±101.6 cm^3^) and heavier (1254.3±158.5 mg) than control tumors (478.2±88.5 cm^3^ and 524.6±109.7 mg) at the end of experiments (**Figure 7B**). In agreement with NRP-1 expression in cultured cells (**Figure 1D** and **Figure 2B**), MDA-MB-231-NRP^low^ tumors had a weaker expression of NRP-1 as examined by immunohistochemistry (**Figure 7C**). MDA-MB-231-NRP^low^ tumors also had weaker cell proliferation detected by immunohistochemistry with an Ab against Ki-67 (**Figures 7C, D**).

The effects of NRP-1 depletion on liver metastasis of TNBC cells were examined by adopting mouse 4T1 cells, which can spontaneously metastasize from the primary tumors to distant sites including livers (28). Primary 4T1-NRP^low^ tumors were significantly smaller than control 4T1-Sc tumors (**Figure S6** in **Supplementary Material**). 4T1-NRP^low^ cells injected into the mice had formed significantly fewer metastatic nodules (Number: 45.6± 8.5) in livers (with an average number of 45.6± 8.5) than 4T1-Sc cells (Number: 102.7 ±18.6) (**Figures 7E, F**).

### Transfection of miR-124-3p Mimics Represses the Growth of TNBC Tumors by Inhibiting NRP-1 Expression *In Vivo*

**Figure 8:**
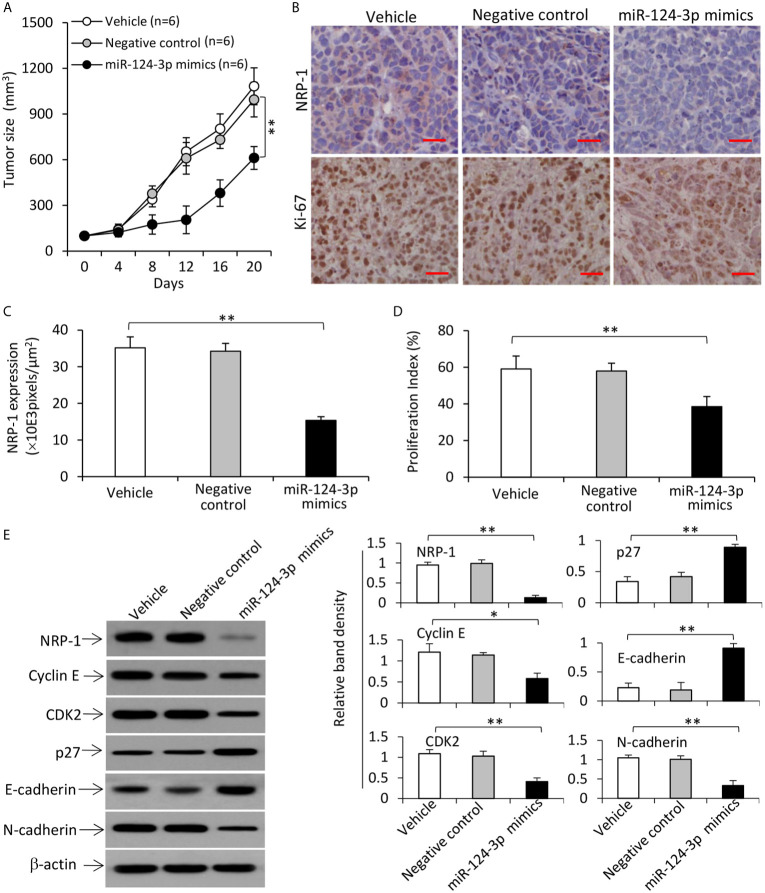
Transfection of miR-124-3p mimics represses the growth of TNBC tumors by downregulating NRP-1 in mice. **(A)** MDA-MB-231 tumors were established in mice, which received intratumoral injections with vehicle, negative control or miR-124-3p mimics oligonucleotides when tumors reached ~100 mm^3^. **(A)** The growth curve of tumors was recorded. **(B)** Illustrated are representative tumor sections prepared 3 days following intratumoral injection, and immunostained brown with Abs against NRP-1 (upper panel) and Ki-67 (lower panel). Magnification bar = 100 µm. **(C)** NRP-1 expression was expressed as pixels/μm^2^ as analyzed by ImageJ, and **(D)** the proliferation index was calculated by counting Ki-67-positive cells. **(E)** Tumor homogenates were subjected to Western blot analysis. Band density was normalized to β-actin. “n”, number of mice. “*P < 0.05”; “**P < 0.001” (one-way ANOVA with a Tukey post-hoc test).

Tumors treated with miR-124-3p mimics were significantly smaller (611.6±74.8 mm^3^) than vehicle-injected tumors (1082.4 ± 120.5 mm^3^), which is not significantly different from negative control-treated tumors (994.7±113.4 mm^3^), 20 days after treatment commencement (**Figure 8A**). The downregulation of NRP-1 and reduced expression of Ki-67 in miR-124-3p mimics-treated tumors harvested 3 days after gene injection were confirmed by immunohistochemistry (**Figures 8B–D**). Western blot analysis of tumor homogenates also showed downregulation of NRP-1, cyclin E, CDK2 and N-cadherin, and upregulation of p27 and E-cadherin in miR-124-3p mimics-treated tumors (**Figure 8E**).

### NRP-1 Co-Activates the TGF-β Signaling Pathway Through Binding With TGF-β

**Figure 9:**
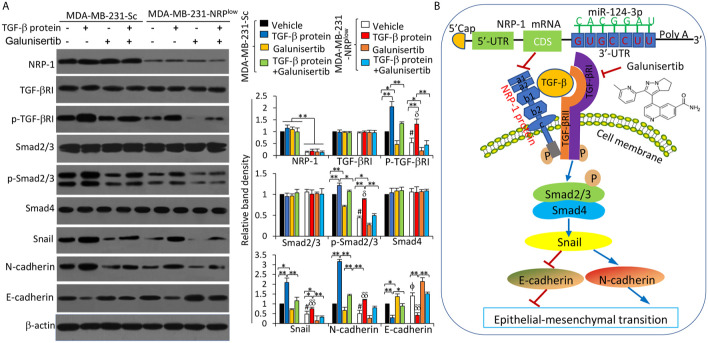
The miR-124-3p/NRP-1 axis co-activates the TGF-β signaling pathway. **(A)** MDA-MB-231-Sc and MDA-MB-231-NRP^low^ cells were treated with recombinant TGF-β protein (5 ng/ml), or galunisertib (10 μg/ml) or the combination for 24 h. Western blot analysis was used to detect the expression of proteins in cell lysates. Band density was measured and normalized to the band of each protein from vehicle-treated MDA-MB-231-Sc cells, which was set to 1. A one-way ANOVA with a Tukey post-hoc test was used for statistical analysis. “*P < 0.05” and “**P < 0.001” indicate a significant difference; “^ϕ^P < 0.05” indicates a significant increase, while “^#^P < 0.05”, a significant reduction, from vehicle-treated MDA-MB-231-Sc cells; “^δ^P < 0.05” and “^δδ^P < 0.001” indicate a significant difference from TGF-β-treated MDA-MB-231-Sc cells. **(B)** A proposed diagram shows how the miR-124-3p/NRP-1 axis co-activates the TGF-β signaling pathway to regulate EMT-related molecules in TNBC cells. MiR-124-3p binds to the 3’-UTR of NRP-1 mRNA and inhibits NRP-1 expression. The structure of NRP-1 protein contains 5 extracellular domains, a transmembrane domain and a short cytosolic tail. “→” indicates promoting, or activating, while “⟘“, inhibiting or blocking. “p” indicates protein phosphorylation. NRP-1, neuropilin-1; CDS, coding sequence; TGF-β, transforming growth factor-β, TGF-βR, TGF-β receptor; UTR, untranslated region.

It has been reported that NRP-1 can co-activate the TGF-β pathway (29), which plays a key role in the progression of TNBC (30). We confirmed the binding of TGF-β and NRP-1 in MDA-MB-231 cells as detected by co-immunoprecipitation assay (**Figure S7** in **Supplementary Material**), in agreement with published studies (8, 31). We also showed that the interaction of NRP-1 and TGF-β ligand-induced the activation of the TGF-β pathway in MDA-MB-231 cells. Recombinant human TGF-β protein, a stimulator of the TGF-β pathway, and galunisertib, which is a specific inhibitor of TGF-β receptor (TGF-βR) and has been tested to treat breast cancer in clinical trials (32), or their combination were added to cultured cells. TGF-β protein and/or galunisertib had little effect on the expression of NRP-1 or TGF-βRI (**Figure 9A**). However, recombinant TGF-β protein increased, while galunisertib reduced, the expression of p-TGF-βRI. The activation of the TGF-β signaling pathway by TGF-β protein, or the inhibition by galunisertib or NRP-1 depletion, had no effect on the expression of Smad2/3 or Samd4. However, TGF-β protein incubation induced an increase in p-Smad2/3, Snail and N-cadherin expressions, and reduced the expression of E-cadherin; while inhibition of TGF-β signaling by galunisertib or NRP-1 depletion reduced the expression of p-Smad2/3, Snail and N-cadherin, and increased the expression of E-cadherin, and also abolished the effects of TGF-β protein in part (**Figure 9A**).

## Discussion

NRP-1 is involved in the progression of various cancer types by co-activating multiple cognate receptor tyrosine kinase signaling pathways (6, 7, 23). In breast cancer, particularly TNBC, NRP-1 is an isoform-specific receptor for VEGF and the VEGF/NRP-1 axis promotes cell proliferation and migration by increasing the activity of cell division control protein 42 homolog (Cdc42) (14) or enhancing the EMT and activation of NF-κB and β-catenin (15). In accord, the present study has demonstrated that NRP-1 acts as a co-receptor for TGF-β to activate the TGF-β pathway in TNBC cells. We have also identified miR-124-3p as an upstream regulator for NRP-1 by binding to its 3’-UTR in TNBC cells (**Figure 9B**). Some miRNAs, such as miRNA-148, miR-124, miR-320 and miR-141, have been shown to negatively regulate the expression of NRP-1 in hepatocellular carcinoma (HCC) (21), glioma (22), cholangiocarcinoma (23) and pancreatic ductal carcinoma (7), respectively, supporting the theory that one target gene can be regulated by multiple miRNAs (17). To our knowledge, the present study may be the first one revealing a regulatory link between miR-124-3p and NRP-1 and elucidating some of the functional role of the miR-124-3p/NRP-1 axis in the proliferation and metastasis of TNBC cells.

MiR-124 is one of the most studied miRNAs because it is downregulated and contributes to the development, progression and prognosis in various human malignancies (33). In a study with miRNA library screening with functional proteomics and integrated analysis of clinical data, miR-124 has been identified to be a potential suppressor capable of reducing p27 expression by binding to the promoter region, leading to a subsequent G1 arrest and inhibiting the invasive ability of breast cancer cells (18). In the present study, we have demonstrated that miR-124-3p reduced the expression of p27 through downregulating NRP-1. It has been reported that NRP-1, by co-activating the HGF/c-Met pathway (6), increases the phosphorylation of Akt (34), which in turn leads to p27 downregulation, inhibiting the activation of the CDK2/cyclin E complex, resulting in sequential cell cycle arrest (35). However, other studies suggest that miR-124-3p exerts a suppressive function by targeting different genes. For example, miR-124 downregulates PIK3CA (phosphatidylinositol-4,5-bisphosphate 3-kinase catalytic subunit alpha), suppressing the proliferation of HCC cells (36); miR-124-3p inhibits the invasiveness and metastasis of HCC cells by targeting CRKL (Crk-like protein) (37); and miR-124-3p regulates FGF2 (fibroblast growth factor 2)-EGFR pathway to overcome pemetrexed resistance in lung adenocarcinoma cells by targeting MGAT5 (alpha-1,6-mannosylglycoprotein 6-beta-N-acetylglucosaminyltransferase) (38). These results suggest that the role of miR-124-3p is complex, and cell- and disease-dependent.

Numerous studies indicate that EMT is pivotal for the invasion and metastasis of TNBC cells (39). Here we have shown that NRP-1 binds with TGF-β to co-activate the TGF-β pathway and promote EMT in TNBC cells (**Figure 9B**). In support, it has been reported that NRP-1 serving as a high-affinity receptor for TGF-βcan activate the TGF-β signaling in MDA-MB-231 cells (8, 31). Interestingly, NRP-1 has also been reported to contribute to TGF−β−induced EMT and metastasis of non−small cell lung cancer cells by binding with TGFβRII (40). In the TGF−β signaling pathway, TGF−β binds to TGFβRII on the cell membrane to recruit TGFβRI and form a complex, leading to the phosphorylation of TGFβRI (41). On the other hand, NRP-1 depletion or inhibition of TGF-β signaling by galunisertib could inhibit the phosphorylation of TGFβRI (**Figure 9B**). Therefore, the interaction of NRP-1 with the TGF-β pathway may be conducted through binding with different parts of the TGF−β complex in different cell types. The phosphorylated TGFβRI can, in turn, induce the phosphorylation of Smad2/3 that forms a trimeric SMAD with Smad4, leading to the upregulation of Snail, which conveys TGF-β-induced repression of N-cadherin upregulation and E-cadherin downregulation, the two well-known hallmarks of EMT (41) (**Figure 9B**). EMT is also associated with the increased expression and activities of MMP-2 and MMP-9, which can stimulate the metastasis of cancer cells (42).

Although the regulatory effect of miR-124-3p on NRP-1 expression and their roles on the proliferation and metastasis of human and mouse TNBC cells have been evaluated in the study, the molecular expression and interaction have not been fully validated in mouse 4T1 cells due to the unavailability of specific antibodies. Further investigations on this point by adopting mouse TNBC cells, and maybe other human cell lines, can demonstrate the reproducibility in phenotypes and confirm the generalizability of the miR-124-3p/NRP-1 axis in TNBC cells.

We have previously reported that the miR-141/NRP-1 axis was associated with clinicopathology and contributed to the growth and metastasis of pancreatic cancer (7). Unfortunately, we have not validated the significance of the miR-124-3p/NRP-1 axis in clinical TNBC data. Such a study may further confirm the role of this axis by analyzing the association of expression levels of miR-124-3p and NRP-1 with clinicopathological parameters and survival data of human TNBC. It has been reported that the expression level of NRP-1 correlates with lymph metastasis (13, 15) and inversely correlates with the survival in breast cancer (16). A recent study suggests that NRP-1 may be an independent prognostic factor for TNBC patients (43) and increased NRP-1 expression has been observed after neoadjuvant chemotherapy in breast cancer patients (44). All these studies highlight the significance and importance of NRP-1 in breast cancer, particularly, TNBC.

TNBC cells, such as MDA-MB-231 and MDA-MB-453 cells, have been shown to express higher levels of NRP-1 than non-TNBC MCF-7 and ZR-75-1 cells (45, 46). The present study has confirmed this finding that TNBC cells expressed higher levels of NRP-1 and lower levels of miR-124-3p than non-TNBC cells. The higher expression of NRP-1 has also been found in TNBC tumor tissues (16). TNBC is characterized by lacking the expression of estrogen receptor, progesterone receptor, and HER-2 (2). Therefore, the intrinsic molecular linkage of these three receptors, particularly, HER-2, with the miR-124-3p/NRP-1 axis may be worth further investigation for elucidating the underlying mechanisms.

In summary, TNBC only accounts for around 15% of all breast cancers but it is responsible for about 90% of breast cancer-associated mortality (2, 3). The lack of effective therapeutics highlights the need for further exploring the underlying mechanisms contributing to its aggressive features. The function of the miR-124-3p/NRP-1 axis involved in the proliferation and metastasis of TNBC cells suggests that these molecules may be potential therapeutic targets and valuable biomarkers for TNBC and warrant further investigation.

## Data Availability Statement

The original contributions presented in the study are included in the article/**Supplementary Material**. Further inquiries can be directed to the corresponding authors.

## Ethics Statement

The animal study was reviewed and approved by the Animal Ethics Committee of Harbin Medical Unversity.

## Author Contributions

XS and XJ designed the study. JZ and XZ performed the majority of the experiments and data analysis. ZL and YS assisted with the *in vivo* experiments. JZ and XS drafted the manuscript. JZ and XZ contributed equally to this work. All authors contributed to the article and approved the submitted version.

## Funding

This work was supported in part by grants of National Key Research and Development Program of China (2017YFC1308602), Supportive Funds by Heilongjiang Provincial Department of Science and Technology (GX18C010), National Natural Scientific Foundation of China (81703055), and the Fundamental Research Funds for Heilongjiang Provincial Universities (2017LCZX06).

## Conflict of Interest

The authors declare that the research was conducted in the absence of any commercial or financial relationships that could be construed as a potential conflict of interest.

## Abbreviations

ANOVA, analysis of variance; BrdU, bromodeoxyuridine; CDK2, cyclin-dependent kinase 2; CDS, coding sequence; DMEM, Dulbecco’s Modified Eagle Medium; EGF, epidermal growth factor; EMT, epithelial-mesenchymal transition; FBS, fetal bovine serum; HER-2, human epidermal growth factor receptor-2; HE, hematoxylin and eosin; HGF, hepatic growth factor; HMEpC, human mammary epithelial cells; miRNA, microRNA; MMP, matrix metalloproteinase; NRP-1, neuropilin-1; PI3K, phosphoinositide 3-kinase; qRT-PCT, quantitative reverse-transcription polymerase chain reaction; TGF-β, transforming growth factor-β; TGF-βR, TGF-β receptor; TNBC, triple-negative breast cancer; UTR, untranslated region; VEGF, vascular endothelial growth factor.

## References

[1] Siegel RL Miller KD Jemal A. Cancer statistics, 2020. CA: Cancer J Clin (2020) 70:7–30. 10.3322/caac.21590 31912902

[2] Lee JS Yost SE Yuan Y. Neoadjuvant Treatment for Triple Negative Breast Cancer: Recent Progresses and Challenges. Cancers (2020) 12:1404. 10.3390/cancers12061404 PMC7352772 32486021

[3] Staaf J Glodzik D Bosch A Vallon-Christersson J Reuterswärd C Häkkinen J. Whole-genome sequencing of triple-negative breast cancers in a population-based clinical study. Nat Med (2019) 25:1526–33. 10.1038/s41591-019-0582-4 PMC6859071 31570822

[4] Hamerlik P Lathia JD Rasmussen R Wu Q Bartkova J Lee M. Autocrine VEGF-VEGFR2-Neuropilin-1 signaling promotes glioma stem-like cell viability and tumor growth. J Exp Med (2012) 209:507–20. 10.1084/jem.20111424 PMC3302235 22393126

[5] Rizzolio S Rabinowicz N Rainero E Lanzetti L Serini G Norman J. Neuropilin-1-dependent regulation of EGF-receptor signaling. Cancer Res (2012) 72:5801–11. 10.1158/0008-5472.CAN-12-0995 22986738

[6] Li L Jiang X Zhang Q Dong X Gao Y He Y. Neuropilin-1 is associated with clinicopathology of gastric cancer and contributes to cell proliferation and migration as multifunctional co-receptors. J Exp Clin Cancer Res CR (2016) 35:16. 10.1186/s13046-016-0291-5 26795388 PMC4722781

[7] Ma L Zhai B Zhu H Li W Jiang W Lei L. The miR-141/neuropilin-1 axis is associated with the clinicopathology and contributes to the growth and metastasis of pancreatic cancer. Cancer Cell Int (2019) 19:248. 10.1186/s12935-019-0963-2 31572065 PMC6764122

[8] Glinka Y Stoilova S Mohammed N Prud’homme GJ. Neuropilin-1 exerts co-receptor function for TGF-beta-1 on the membrane of cancer cells and enhances responses to both latent and active TGF-beta. Carcinogenesis (2011) 32:613–21. 10.1093/carcin/bgq281 21186301

[9] Dent SF. The role of VEGF in triple-negative breast cancer: where do we go from here? Ann Oncol Off J Eur Soc Med Oncol / ESMO (2009) 20:1615–7. 10.1093/annonc/mdp410 19690059

[10] Wendt MK Williams WK Pascuzzi PE Balanis NG Schiemann BJ Carlin CR. he antitumorigenic function of EGFR in metastatic breast cancer is regulated by expression of Mig6. Neoplasia (New York NY) (2015) 17:124–33. 10.1016/j.neo.2014.11.009 PMC4309683 25622905

[11] Parr C Watkins G Mansel RE Jiang WG. The hepatocyte growth factor regulatory factors in human breast cancer. Clin Cancer Res (2004) 10:202–11. 10.1158/1078-0432.CCR-0553-3 14734471

[12] Pang MF Georgoudaki AM Lambut L Johansson J Tabor V Hagikura K. TGF-β1-induced EMT promotes targeted migration of breast cancer cells through the lymphatic system by the activation of CCR7/CCL21-mediated chemotaxis. Oncogene (2016) 35:748–60. 10.1038/onc.2015.133 PMC4753256 25961925

[13] Seifi-Alan M Shams R Bandehpour M Mirfakhraie R Ghafouri-Fard S. Neuropilin-1 expression is associated with lymph node metastasis in breast cancer tissues. Cancer Manage Res (2018) 10:1969–74. 10.2147/CMAR.S169533 PMC6045910 30022855

[14] Kiso M Tanaka S Saji S Toi M Sato F. Long isoform of VEGF stimulates cell migration of breast cancer by filopodia formation via NRP1/ARHGAP17/Cdc42 regulatory network. Int J cancer. J Int Cancer (2018) 143:2905–18. 10.1002/ijc.31645 PMC6282968 29971782

[15] Luo M Hou L Li J Shao S Huang S Meng D. VEGF/NRP-1axis promotes progression of breast cancer via enhancement of epithelial-mesenchymal transition and activation of NF-κB and β-catenin. Cancer Lett (2016) 373:1–11. 10.1016/j.canlet.2016.01.010 26805761

[16] Naik A Al-Zeheimi N Bakheit CS Al Riyami M Al Jarrah A Al Moundhri MS. Neuropilin-1 Associated Molecules in the Blood Distinguish Poor Prognosis Breast Cancer: A Cross-Sectional Study. Sci Rep (2017) 7:3301. 10.1038/s41598-017-03280-0 28607365 PMC5468252

[17] Bartel DP. MicroRNAs: genomics, biogenesis, mechanism, and function. Cell (2004) 116:281–97. 10.1016/S0092-8674(04)00045-5 14744438

[18] Seviour EG Sehgal V Lu Y Luo Z Moss T Zhang F. Functional proteomics identifies miRNAs to target a p27/Myc/phospho-Rb signature in breast and ovarian cancer. Oncogene (2016) 35:691–701. 10.1038/onc.2014.469 25639871 PMC4522411

[19] McGuire A Brown JA Kerin MJ. Metastatic breast cancer: the potential of miRNA for diagnosis and treatment monitoring. Cancer Metastasis Rev (2015) 34:145–55. 10.1007/s10555-015-9551-7 PMC4368851 25721950

[20] Ma L Teruya-Feldstein J Weinberg RA. Tumour invasion and metastasis initiated by microRNA-10b in breast cancer. Nature (2007) 449:682–8. 10.1038/nature06174 17898713

[21] Liu Q Xu Y Wei S Gao W Chen L Zhou T. miRNA-148b suppresses hepatic cancer stem cell by targeting neuropilin-1. Biosci Rep (2015) 35:e00229. 10.1042/BSR20150084 25997710 PMC4613672

[22] Zhang G Chen L Khan AA Li B Gu B Lin F. miRNA-124-3p/neuropilin-1(NRP-1) axis plays an important role in mediating glioblastoma growth and angiogenesis. Int J cancer. J Int du Cancer (2018) 143:635–44. 10.1002/ijc.31329 29457830

[23] Zhu H Jiang X Zhou X Dong X Xie K Yang C. Neuropilin-1 regulated by miR-320 contributes to the growth and metastasis of cholangiocarcinoma cells. Liver Int Off J Int Assoc Study Liver (2018) 38:125–35. 10.1111/liv.13495 28618167

[24] Wei Z Jiang X Qiao H Zhai B Zhang L Zhang Q. STAT3 interacts with Skp2/p27/p21 pathway to regulate the motility and invasion of gastric cancer cells. Cell Signall (2013) 25:931–8. 10.1016/j.cellsig.2013.01.011 23333463

[25] Zhai B Hu F Jiang X Xu J Zhao D Liu B. Inhibition of Akt reverses the acquired resistance to sorafenib by switching protective autophagy to autophagic cell death in hepatocellular carcinoma. Mol Cancer Ther (2014) 13:1589–98. 10.1158/1535-7163.MCT-13-1043 24705351

[26] Han P Li H Jiang X Zhai B Tan G Zhao D. Dual inhibition of Akt and c-Met as a second-line therapy following acquired resistance to sorafenib in hepatocellular carcinoma cells. Mol Oncol (2017) 11:320–34. 10.1002/1878-0261.12039 PMC5527443 28164434

[27] Lourenco AR Ban Y Crowley MJ Lee SB Ramchandani D Du W. Differential Contributions of Pre- and Post-EMT Tumor Cells in Breast Cancer Metastasis. Cancer Res (2020) 80:163–9. 10.1158/0008-5472.CAN-19-1427 PMC6980649 31704888

[28] Yang S Zhang JJ Huang XY. Mouse models for tumor metastasis. Methods Mol Biol (Clifton NJ) (2012) 928:221–8. 10.1007/978-1-62703-008-3_17 PMC3674868 22956145

[29] Vivekanandhan S Mukhopadhyay D. Genetic status of KRAS influences Transforming Growth Factor-beta (TGF-beta) signaling: An insight into Neuropilin-1 (NRP1) mediated tumorigenesis. Semin Cancer Biol (2019) 54:72–9. 10.1016/j.semcancer.2018.01.014 PMC6072630 29409705

[30] Bhola NE Balko JM Dugger TC Kuba MG Sánchez V Sanders M. TGF-β inhibition enhances chemotherapy action against triple-negative breast cancer. J Clin Invest (2013) 123:1348–58. 10.1172/JCI65416 PMC3582135 23391723

[31] Glinka Y Prud’homme GJ. Neuropilin-1 is a receptor for transforming growth factor beta-1, activates its latent form, and promotes regulatory T cell activity. J Leukocyte Biol (2008) 84:302–10. 10.1189/jlb.0208090 PMC2504713 18436584

[32] Herbertz S Sawyer JS Stauber AJ Gueorguieva I Driscoll KE Estrem ST. Clinical development of galunisertib (LY2157299 monohydrate), a small molecule inhibitor of transforming growth factor-beta signaling pathway. Drug Des Dev Ther (2015) 9:4479–99. 10.2147/DDDT.S86621 PMC4539082 26309397

[33] Jia X Wang X Guo X Ji J Lou G Zhao J. MicroRNA-124: An emerging therapeutic target in cancer. Cancer Med (2019) 8:5638–50. 10.1002/cam4.2489 PMC6745873 31389160

[34] Birchmeier C Birchmeier W Gherardi E Vande Woude GF. Met, metastasis, motility and more. Nat Rev Mol Cell Biol (2003) 4:915–25. 10.1038/nrm1261 14685170

[35] Wander SA Zhao D Slingerland JM. p27: a barometer of signaling deregulation and potential predictor of response to targeted therapies. Clin Cancer Res (2011) 17:12–8. 10.1158/1078-0432.CCR-10-0752 PMC3017239 20966355

[36] Lang Q Ling C. MiR-124 suppresses cell proliferation in hepatocellular carcinoma by targeting PIK3CA. Biochem Biophys Res Commun (2012) 426:247–52. 10.1016/j.bbrc.2012.08.075 22940133

[37] Majid A Wang J Nawaz M Abdul S Ayesha M Guo C. miR-124-3p Suppresses the Invasiveness and Metastasis of Hepatocarcinoma Cells via Targeting CRKL. Front Mol Biosci (2020) 7:223. 10.3389/fmolb.2020.00223 33094104 PMC7522612

[38] Cai J Huang J Wang W Zeng J Wang P. miR-124-3p Regulates FGF2-EGFR Pathway to Overcome Pemetrexed Resistance in Lung Adenocarcinoma Cells by Targeting MGAT5. Cancer Manage Res (2020) 12:11597–609. 10.2147/CMAR.S274192 PMC7674808 33223850

[39] Khaled N Bidet Y. New Insights into the Implication of Epigenetic Alterations in the EMT of Triple Negative Breast Cancer. Cancers (2019) 11:559. 10.3390/cancers11040559 PMC6521131 31003528

[40] Ding Z Du W Lei Z Zhang Y Zhu J Zeng Y. Neuropilin 1 modulates TGF−β1−induced epithelial−mesenchymal transition in non−small cell lung cancer. Int J Oncol (2020) 56:531–43. 10.21203/rs.2.10348/v1 PMC6959462 31894269

[41] Vincent T Neve EP Johnson JR Kukalev A Rojo F Albanell J. A SNAIL1-SMAD3/4 transcriptional repressor complex promotes TGF-beta mediated epithelial-mesenchymal transition. Nat Cell Biol (2009) 11:943–50. 10.1038/ncb1905 PMC3769970 19597490

[42] Lamouille S Xu J Derynck R. Molecular mechanisms of epithelial-mesenchymal transition. Nat Rev Mol Cell Biol (2014) 15:178–96. 10.1038/nrm3758 PMC4240281 24556840

[43] Wang H Zhang YN Xu DQ Huang JG Lv D Shi XY. Neuropilin1, a novel independent prognostic factor and therapeutic target in triple-negative breast cancer. Neoplasma (2020) 67:1335–42. 10.4149/neo_2020_191127N1223 32657612

[44] Al-Zeheimi N Naik A Bakheit CS Al Riyami M Al Ajarrah A Al Badi S. Neoadjuvant Chemotherapy Alters Neuropilin-1, PlGF, and SNAI1 Expression Levels and Predicts Breast Cancer Patients Response. Front Oncol (2019) 9:323. 10.3389/fonc.2019.00323 31106153 PMC6494932

[45] Sflomos G Dormoy V Metsalu T Jeitziner R Battista L Scabia V. A Preclinical Model for ERα-Positive Breast Cancer Points to the Epithelial Microenvironment as Determinant of Luminal Phenotype and Hormone Response. Cancer Cell (2016) 29:407–22. 10.1016/j.ccell.2016.02.002 26947176

[46] Zhang L Chen Y Li C Liu J Ren H Li L. RNA binding protein PUM2 promotes the stemness of breast cancer cells via competitively binding to neuropilin-1 (NRP-1) mRNA with miR-376a. Biomed Pharmacother = Biomed Pharmacother (2019) 114:108772. 10.1016/j.biopha.2019.108772 30909144

