# Supplementary material for: The miR-124-3p/Neuropilin-1 Axis Contributes to the Proliferation and Metastasis of Triple-Negative Breast Cancer Cells and Co-Activates the TGF-β Pathway
Source: Front Oncol. 2021 Apr 12;11:654672. doi: 10.3389/fonc.2021.654672 (PMC8072051; doi:10.3389/fonc.2021.654672)

Supplementary Material for

The miR-124-3p/neuropilin-1 axis contributes to the proliferation and metastasis of triple-negative breast cancer cells and co-activates the TGF-β pathway

Jiayang Zhang,[1] Xuesong Zhang,[2] Ziyi Li,[3] Qingshan Wang,[2,3] Yan Shi,[4] Xian Jiang,[3,*] Xueying Sun[3,*]

[1]Key Laboratory of Carcinogenesis and Translational Research (Ministry of Education), Department of Breast Oncology, Peking University Cancer Hospital & Institute, Beijing 100142, China.

[2]Department of General Surgery, Heilongjiang Provincial Hospital, Harbin Institute of Technology, Harbin 150036, China.

[3]The Hepatosplenic Surgery Center, the First Affiliated Hospital of Harbin Medical University, Harbin 150001, China.

[4]Department of Pathology, The Second Affiliated Hospital of Harbin Medical University, Harbin 150086, China.

*To whom correspondence should be addressed. Email: sunxueying@hrbmu.edu.cn and jiangxian@hrbmu.edu.cn

Contents

Supplementary Materials and Methods 3

Table S1. List of antibodies, main reagents and kits..3

Table S2. Sequences of miRNA mimics, antagomiR-124-3p and negative control oligonucleotides…………………………………………………………………....4

Transfection of oligonucleotides targeting miRNA-124-3p……………………......4

Cell viability analysis...5

Bromodeoxyuridine incorporation assay……..5

Assessment of cell cycle……………………..6

Transwell migration assay...6

Cell scratch assay………………………………………………………………...6

Quantitative reverse-transcription polymerase chain reaction (qRT-PCR)................7

Western blot analysis..8

Gelatin zymography assay...8

Co-immunoprecipitation assay...9

Immunocytochemistry…………………………………………………..………..10

Immunohistochemistry and in situ Ki-67 proliferation index………...………....10

References...11

Supplementary Figures...11

Figure S1..11

Figure S2..12

Figure S3..12

Figure S4..13

Figure S5..13

Figure S6..14

Figure S7..14

Entire images of Western blots in Figures 1, 2, 4, 5, 6, 8 and 9.............................15

Supplementary Materials and Methods

Table S1. List of antibodies, reagents and kits

Name	Catalogue No.	Supplier
Anti-Snail Ab	#3879	CST
Anti-E-Cadherin Ab	#3195	CST
Anti- N-Cadherin Ab	#13116	CST
Anti- Smad2/3 Ab	#8685	CST
Anti-phospho-Smad2 (Ser465/467)/ Smad3 (Ser423/425) Ab	#9510	CST
Anti-Smad4 Ab	#38454	CST
Anti-p27 Ab	#3688	CST
Anti-NRP-1 Ab	#3725	CST
Rat FITC-Conjugated Ab	#56722	CST
Anti-β-actin Ab	sc-130065	Santa Cruz
Anti-NRP-1 Ab	sc-5307	Santa Cruz
Anti-cyclin E Ab	sc-247	Santa Cruz
Anti-CDK2 Ab	sc-6248	Santa Cruz
Anti-p21 Ab	sc-53870	Santa Cruz
Anti-cyclin D1	sc-8396	Santa Cruz
Anti-MMP-2	sc-13594	Santa Cruz
Anti-MMP-9	sc-21733	Santa Cruz
Mouse FITC-conjugated Ab	sc-516140	Santa Cruz
Anti-Ki67 Ab	ab15580	Abcam
Anti-TGF-βR I Ab	ab31013	Abcam
Anti- phosphor- TGF-βRI (Ser165) Ab	ab112095	Abcam
Recombinant human TGF-βR 1 protein	ab50036	Abcam
Anti-TGF-β Ab	ab215715	Abcam
HRP goat anti-mouse Ab	TA130004	OriGene
HRP goat anti-rabbit Ab	TA140003	OriGene
HRP rabbit anti-goat Ab	TA130032	OriGene
HRP rabbit anti-rat Ab	TA130038	OriGene
VECTASTAIN® ELITE ABC kit	SK-4100	Vector
Galunisertib	S2230	Selleckchem
TUNEL kit	# 11684795910	Sigma-Aldrich
Lipofectamine2000	#11668019	Invitrogen
CCK-8	CK04-05	Dojindo
DAPI	d9564	Sigma-Aldrich
TRIzol™ Reagent	15596026	ThermoFisher

Note: Ab, primary antibody; CST, Cell Signaling Technology (Boston, MA, USA); Santa Cruz; Santa Cruz Biotechnology (Santa Cruz, CA, USA); Cytoskeleton (Denver, CO, USA); R&D Systems (Minneapolis, MN, USA); Sigma-Aldrich (St. Louis, MO, USA); Invitrogen (Carlsbad, CA, USA),

MMP-2, matrix metalloproteinase-2; MMP-9, matrix metalloproteinase-9; FITC, fluorescein isothiocyanate; HRP, horseradish peroxidase; OriGene (OriGene Technologies, Inc., Beijing, China); TUNEL, Terminal deoxynucleotidyl transferase-mediated dUTP nick-end labeling agent; Dojindo, Dojindo Molecular Technologies, Gaithersburg, MD, USA; Vector, Vector Laboratories (CA, USA); DAPI, 4',6-diamidino-2-phenylindole.

Table S2. Sequences of miRNA mimics, antagomiR-124-3p and negative control oligonucleotides

Oligonucleotides		Sequences
**Mimics**	miR-24	5′-UCCGGUGCCUACUGAGCUGAUAUCAGUUCUCAUUUUACACACUGG CUCAGUUCAGCAGGAACAGGAG-3′
	miR-30c	5′-CCAUGCUGUAGUGUGUGUAAACAUCCUACACUCUCAGCUGUGAGC UCAAGGUGGCUGGGAGAGGGUUGUUUACUCCUUCUGCCAUGGA-3′
	miR-124-3p	5′-CUCUCUCCGUGUUCACAGCGGACCUUGAUUUAAAUGUCCAUACAA UUAAGGCACGCGGUGAAUGCCAAGAAUGGGGC-3′
	miR-130a	5′-GGCCAGAGCUCUUUUCACAUUGUGCUACUGUCUGCACCUGUCACU AGCAGUGCAAUGUUAAAAGGGCAUUGGCCG-3′
	miR-141	5′-GGGUCCAUCUUCCAGUACAGUGUUGGAUGGUCUAAUUGUGAAGC UCCUAACACUGUCUGGUAAAGAUGGCUCCCGGG-3′
	miR-148a	5′-GAGGCAAAGUUCUGAGACACUCCGACUCUGAGUAUGAUAGAAGUC AGUGCACUACAGAACUUUGUCUC-3′
	miR-152	5′-GGCCCAGGUUCUGUGAUACACUCCGACUCGGGCUCUGGAGCAGUC AGUGCAUGACAGAACUUGGGCCCGG-3′
	miR-181b	5′-GGUCACAAUCAACAUUCAUUGCUGUCGGUGGGUUGAACUGUGUG GACAAGCUCACUGAACAAUGAAUGCAACUGUGG-3′
	miR-200a	5′-GUGAGCAUCUUACCGGACAGUGCUGGAUUUCCCAGCUUGACUCUA ACACUGUCUGGUAACGAUGUUCAAAGG-3′
	miR-212	5′-GGACAGCGCGCCGGCACCUUGGCUCUAGACUGCUUACUGCCCGGG CCGCCCUCAGUAACAGUCUCCAGUCACGGCCACCG-3′
	miR-320	5′-CCGCCUUCUCUUCCCGGUUCUUCCCGGAGUCGGGAAAAGCUGGGU UGAGAGGGCGAAAAAG-3′
	miR-376b	5′-GGUAUUUAAAACGUGGAUAUUCCUUCUAUGUUUACGUGAUUCCU GGUUAAUCAUAGAGGAAAAUCCAUGUUUUCAG′3
AntagomiR-124-3p		5′-GGCAUUCACCGCGUGCCUUA-3′
Negative control		5′-CAGUACUUUUGUGUAGUACAA-3′

Transfection of oligonucleotides targeting miRNAs

The double-stranded mimics targeting various miRNAs, antagomiR-124-3p and the negative control (NC) oligonucleotides were purchased from GenePharma Co., Ltd.,

Shanghai, China). Cells were grown to 60-70% confluence and incubated with RNAs at a final concentration of 0.1 μM by using Lipofectamine^{TM} 2000 (Invitrogen) in serum-free media for 48 h and then subjected to assays.

Cell viability assay

The Cell Counting Kit-8 (CCK-8) (Dojindo Molecular Technologies, Inc. Beijing, China) was used to determine cell viability. Cells were seeded at 1×10^3 cells /well in 96-well plates. At different time points, the culture medium was replaced with 100 μl of fresh medium containing 10 μl of CCK-8 solution. The cells were further incubated for 2 h at 37°C, and the optical density (OD) at 450 nm was measured. The viability of cells was calculated by using a formula as below: (Experimental OD-Control OD)/Control OD × 100%. The experiment was repeated thrice.

Bromodeoxyuridine incorporation assay

Cell proliferation was detected by using the 5-bromo-2-deoxyuracil (BrdU) labeling and detection kit (Boster Biological Technology Ltd., China) following the manufacturer's protocol. In brief, cells were cultured for a certain time and BrdU reagent was added to the medium at the final concentration of 10 μM and incubated for 60 min. Cells were fixed with 3% formaldehyde and then incubated with an anti-BrdU mAb, followed by a fluorescein-labeled secondary Ab. Cells were counterstained with 4',6-diamidino-2-phenylindole (DAPI) to visualize the nuclei. BrdU-labeled indices were measured by visually scoring nuclei stained with DAPI. BrdU-positive cells were scored as a percentage of the total cell number. The experiment was repeated thrice.

Assessment of cell cycle

Cells were seeded at 5.0×10^5 cells/well in 6-well plates, cultured for 48 h, and then harvested. The percentage of cells at G1 and S phases was determined with a cell cycle detection kit (BD Biosciences, Beijing, China) using flow cytometry with a Beckman Coulter Epics Altra II cytometer (Beckman Coulter, California, USA). The experiments were repeated thrice.

Transwell migration assay

Transwell assays were performed using 8-μm pore transwell chambers in 24-well plates BD Bioscience (San Jose, CA, USA). Cells (1×10^4) suspended in 200 μl of serum-free DMEM/F12 medium were seeded on the polycarbonate membrane in a transwell culture chamber and the lower chamber was filled with 800 μl of medium with 10% FBS. After incubation for 12 or 24 h at 37°C in a humidified atmosphere of 5% CO_2, the transwell culture chamber was washed with PBS and the cells on the top surface of the polycarbonate membrane were removed. Cells that migrated to the bottom surface of the insert were fixed with 4% paraformaldehyde for 15 min and stained with 0.3% crystalline violet for 15 min. The unpenetrated cells were removed with a cotton swab, and cells were counted based on digital images of 5 randomly selected fields under light microscopy.

Matrigel invasion assay

This assay was similar to the transwell migration assay as described above except that the transwell insert membrane was coated with Matrigel while in migration assay it was not. Matrigel was slowly thawed on ice at 4°C and then mixed with cold culture

medium at a ratio of 1:3 thoroughly. The mixture of Matrigel and culture medium was prepared freshly and added to the insert membrane. Cells (1×10^4) suspended in 200 μl of serum-free medium were seeded in a transwell culture chamber. After incubation for 24 h, cells that invaded into the bottom surface of the insert were fixed with 4% paraformaldehyde, stained with Giemsa stain, and counted under light microscopy.

Cell scratch assay

Cells were grown in monolayer in 6-well plates to ~95% confluency and treated with mitomycin C to arrest cell growth. Two straight scratches were made with a pipette tip across each cell monolayer. Floating cells were removed by washing with PBS and fresh media. Eight areas of scratches lines were marked for each cell monolayer and imaged by camera, and the scratch distances were again recorded at indicated time points. The original and resulting areas absent of cells were quantified by ImageJ software. The 8 areas of each well were averaged and variation was further assessed with duplicate wells for each treatment.

Quantitative Reverse-Transcription Polymerase Chain Reaction (qRT-PCR)

RNA isolation and quantitative real-time RT-PCR Total RNA, containing miRNA, was extracted from either tissue samples or transfected cells using TRIzol reagent according to the manufacturer’s instructions. The reverse transcription was conducted by using TaqMan MicroRNA Reverse Transcription Kit reagents and Reverse Transcription Primers (RT primers) and cDNA was synthesized. The reaction mixtures for qRT-PCR were prepared with the primers for NRP-1 (Forward: 5'-GGAGCTACTGGGCTGTGAAG-3'; and Reverse: 5'-ACCGTATGTCGGGAACTCT

G-3') and β-actin (Forward: 5'- AGCGAGCATCCCCCAAAGTT -3'; Reverse: 5'-GGGCACGAAGGCTCATCATT-3'), and analyzed by MX3000P Real-time PCR systems (Stratagen, USA). The expression of mature miR-124-3p was performed as above by using a primer (forward, 5'-GUGUUCACAGCGGACCUUG-3'), and human U6 RNA (with a primer of forward, 5'-CTCGCTTCGGCAGCACA-3') was amplified as an internal control. The reverse primers for U6 and miR-124-3p were the universal primer provided by Takara. Experiments were performed in triplicate, and the data were calculated by ΔΔCt methods.

Western blot analysis

Protein concentrations of cell or tissue lysates were determined using the Bio-Rad protein assay (Bio-Rad, Richmond, CA, USA). Lysates were resolved on SDS-polyacrylamide gels, and the proteins transferred to PVDF membranes, and immunoblotted as previously described [1-4]. The density of each band was measured using the FR200 densitometric analysis program (Shanghai, China). In preliminary experiments, serial dilutions of lysates containing 2.5, 5, 10, 20, 40 or 80 μg of protein were immunoblotted. Band intensities were measured and plotted against protein amounts to generate a standard curve, and the amount of protein for each immunoblot was determined. The quantitative measurement of the Western blot bands was repeated three times.

Gelatin zymography assay

The conditioned medium from an equal number of cells that had been incubated in serum-free medium for 48 h was collected and separated on 10% acrylamide gels

containing 0.1% gelatin (Invitrogen). Gels were incubated in 2.5% Triton X-100 solution at room temperature with gentle agitation to remove SDS, and soaked in reaction buffer (50 mM Tris-HCl, pH 7.5, 150 mM NaCl, 10 mM $CaCl_2$, and 0.5 mM $ZnCl_2$) at 37°C overnight. After the reaction, gels were stained for 1 h with staining solution (0.1% Coomassie Brilliant Blue, 30% methanol, and 10% acetic acid) and then destained in the same solution without Coomassie Brilliant Blue. The gelatinolytic activity of MMP-2 and MMP-9 was visualized as a clear band against a dark background of stained gelatin.

Co-immunoprecipitation assay

MDA-MB-231 cells incubated with recombinant TGF-β protein (5 ng/ml) for 4 h were lysed in the RIPA buffer. Cellular debris was pelleted by centrifugation at 10,000 × g for 10 min at 4° C, and the supernatant was transferred to a fresh tube. An affinity purified rabbit anti-NRP-1 Ab or the control rabbit IgG mixed with Protein A-Agarose was added and incubated overnight at 4°C. The immunoprecipitants were collected by centrifugation at 1,000 × g for 10 min at 4°C. After washing the pellet was resuspended in electrophoresis sample buffer, and the eluted proteins were subjected to Western blot as described above by using the anti-NRP-1 Ab. The membrane was stripped and then re-stained with the anti-TGF-β Ab.

Immunocytochemistry

Cells were fixed with 4% paraformaldehyde and permeabilized with 0.1% Triton X-100 in PBS. Cells were blocked in 10% normal serum for 30 min and then incubated with a primary Ab for 90 min, followed by a FITC-conjugated rat anti-mouse Ab. Cells

were visualized under confocal microscopy.

Immunohistochemistry and *in situ* Ki-67 proliferation index

Frozen tissues were sectioned (5μm), blocked for 2 h, and incubated with anti-human NRP-1 (diluted 1:250) and anti-Ki-67 (diluted 1:800) Abs at 4°C overnight. They were subsequently incubated for 30 min with appropriate secondary Abs using the Ultra-Sensitive TMS-P kit (Zhongshan Co., Beijing, China), and immunoreactivity developed with Sigma FAST DAB (3,3'-diaminobenzidine tetrahydrochloride) and $CoCl_2$ enhancer tablets (Sigma-Aldrich, Shanghai, China). Sections were counterstained with hematoxylin, mounted, and examined by microscopy.

For quantifying the expression of NRP-1 in tumor sections, The ImageJ (Image Processing and Analysis in Java) 1.51v software (National Institutes of Health, USA) was used to quantify the expression of each protein in the immunostained sections according to a previously published report [5]. Five rectangular regions of interest (ROI) were randomly selected in each image (100 ×) for the evaluation. The images were converted to 8 bits, a specific threshold was determined and quantification was performed. Data were expressed as pixels/μm^2.

Ki-67 positive cells in immunostained sections as above were counted in 10 randomly selected × 400 high-power fields under microscopy. The Ki-67 proliferation index was calculated according to the following formula: the number of Ki-67 positive cells/ the total cell count × 100%.

References

1. Jiang, W., et al., *Sodium orthovanadate overcomes sorafenib resistance of hepatocellular carcinoma cells by inhibiting Na(+)/K(+)-ATPase activity and hypoxia-inducible pathways.* Sci Rep, 2018. **8**(1): p. 9706.
2. Han, P., et al., *Dual inhibition of Akt and c-Met as a second-line therapy following acquired resistance to sorafenib in hepatocellular carcinoma cells.* Mol Oncol, 2017. **11**(3): p. 320-334.
3. Li, L., et al., *Neuropilin-1 is associated with clinicopathology of gastric cancer and contributes to cell proliferation and migration as multifunctional co-receptors.* J Exp Clin Cancer Res, 2016. **35**(1): p. 16.
4. Zhai, B., et al., *Arsenic trioxide potentiates the anti-cancer activities of sorafenib against hepatocellular carcinoma by inhibiting Akt activation.* Tumour Biol, 2015. **36**(4): p. 2323-34.
5. Jensen, E.C., *Quantitative analysis of histological staining and fluorescence using ImageJ.* Anat Rec (Hoboken), 2013. **296**(3): p. 378-81.

Supplementary Figures

Figure S1

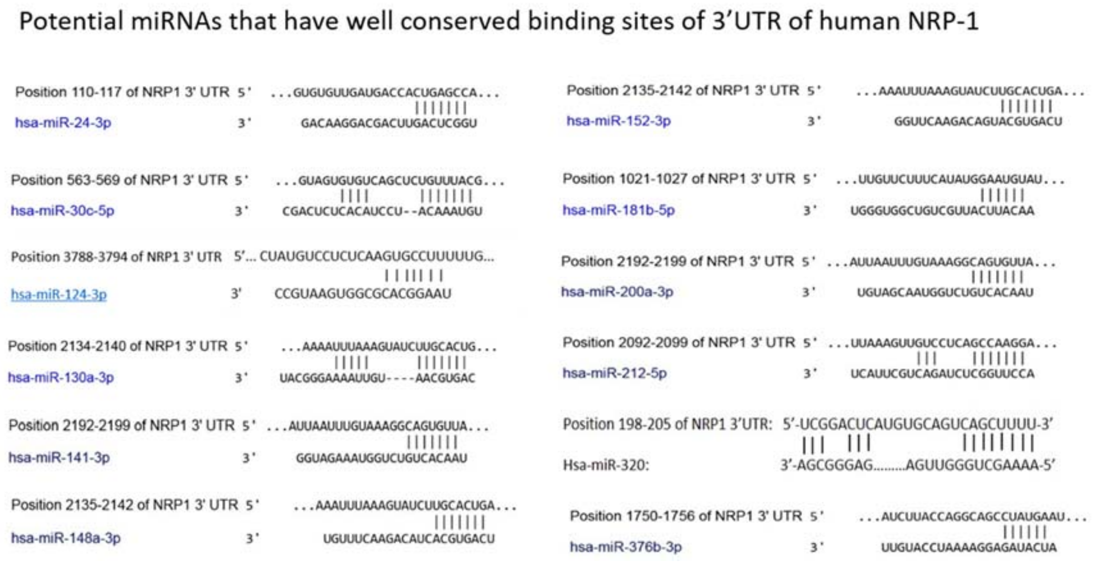

Figure S1. Predicted binding sites of Hsa-miRs to the 3'-UTR of the human NRP-1 gene. Twelve potential miRNAs contain well-conserved binding sites to the 3'-UTR of the human NRP-1 gene as predicted by using miRNA target prediction tools including miRWalk (http://mirwalk.umm.uni-heidelberg.de/), TargetScan (http://www.targetscan.org/), miRanda (https://omictools.com/miranda-tool), miRTarBase (http://mirtarbase.mbc.nctu.edu.tw/) and mirdb (http://mirdb.org/).

Figure S2

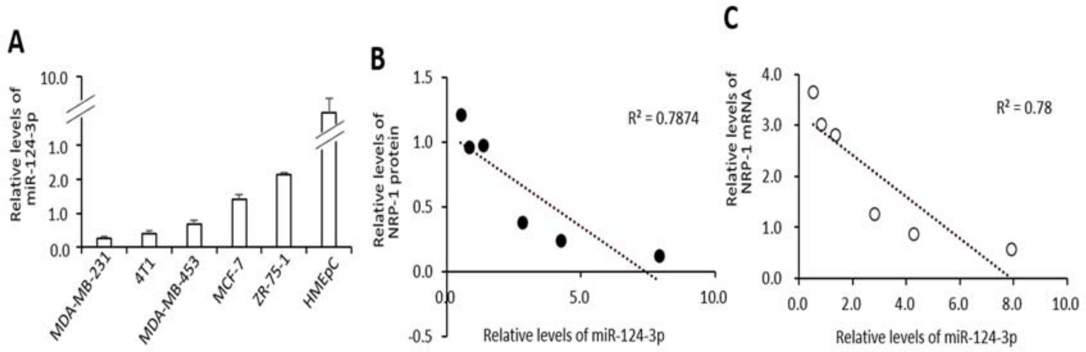

Figure S2. The correlation between miR-124-3p and NRP-1 expression levels in breast cancer cells. (A) A panel of cells were subjected to qRT-PCR for measuring the expression levels of miR-124-3p. (B, C) The correlation of miR-124-3p expression levels with the expression levels of NRP-1 protein (B) and mRNA (C) was analyzed by using a Pearson test, and the correlation coefficient is denoted by "R^2".

Figure S3

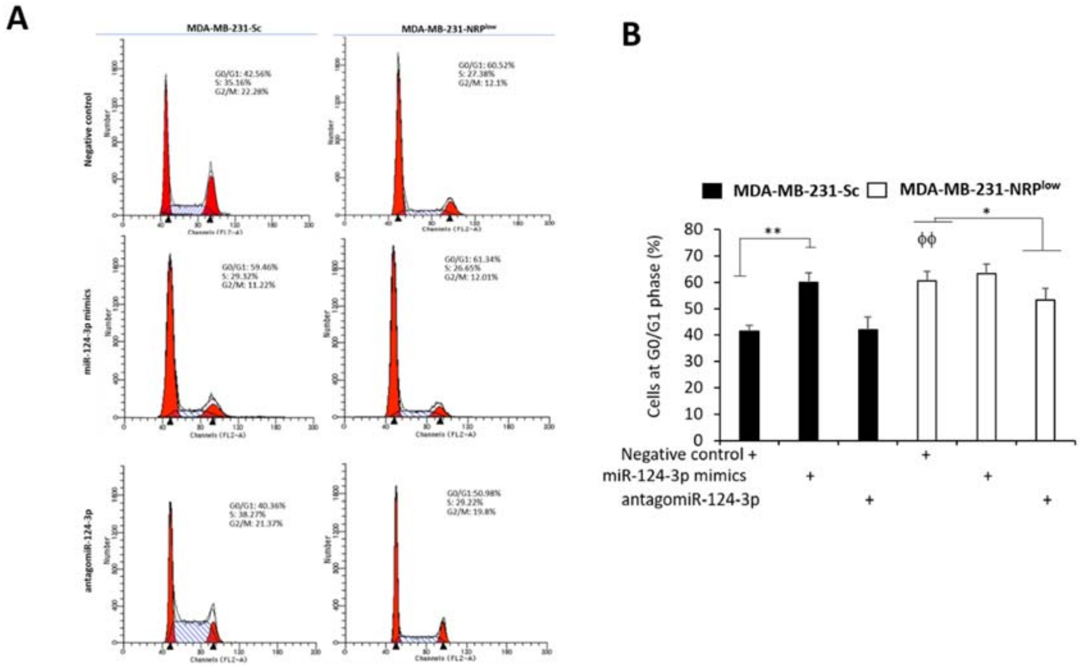

Figure S3. MiR-124-3p inhibits cell proliferation by regulating NRP-1 *in vitro*. MDA-MB-231-Sc and MDA-MB-231-NRP^{low} cells were transfected with negative control, miR-124-3p mimics or antagomiR-124-3p oligonucleotides and continued to be cultured for 48 h. Cells were subjected to flow cytometry for detecting cell cycle distribution (A) and percentages of cells at phase G0/G1 were plotted (B). A one-way ANOVA with a Tukey post-hoc test was used for statistical analysis. "ϕϕ $P<0.001$" indicates a significant increase from negative control-treated MDA-MB-231-Sc cells.

“* P<0.05” and “** P<0.001” indicate a significant difference.

Figure S4

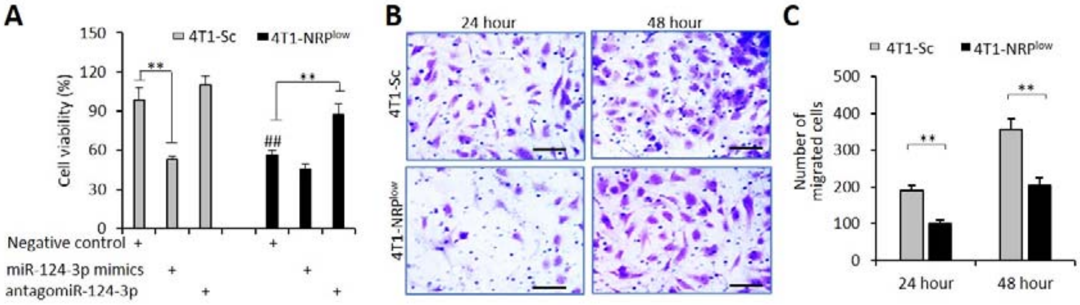

Figure S4. MiR-124-3p inhibits the proliferation and migration of mouse 4T1 cells. 4T1-Sc and 4T1-NRP^{low} cells were transfected with negative control, miR-124-3p mimics or antagomiR-124-3p oligonucleotides for 48 h. (A) The viability of cells was detected by CCK-8 assay and normalized to mock-treated 4T1-Sc cells. (B, C) Transwell migration assays were used to evaluate the migrating ability of the above-transfected cells. Magnification bar = 200 µm. (B) Migrated cells were stained with crystalline violet at 12 and 24 h. A one-way ANOVA with a Tukey post-hoc test was used for statistical analysis. (C) The numbers of migrated cells were counted. “ϕϕ P<0.001” indicates a significant increase from negative control-treated 4T1-Sc cells. “** P<0.001” indicates a significant difference.

Figure S5

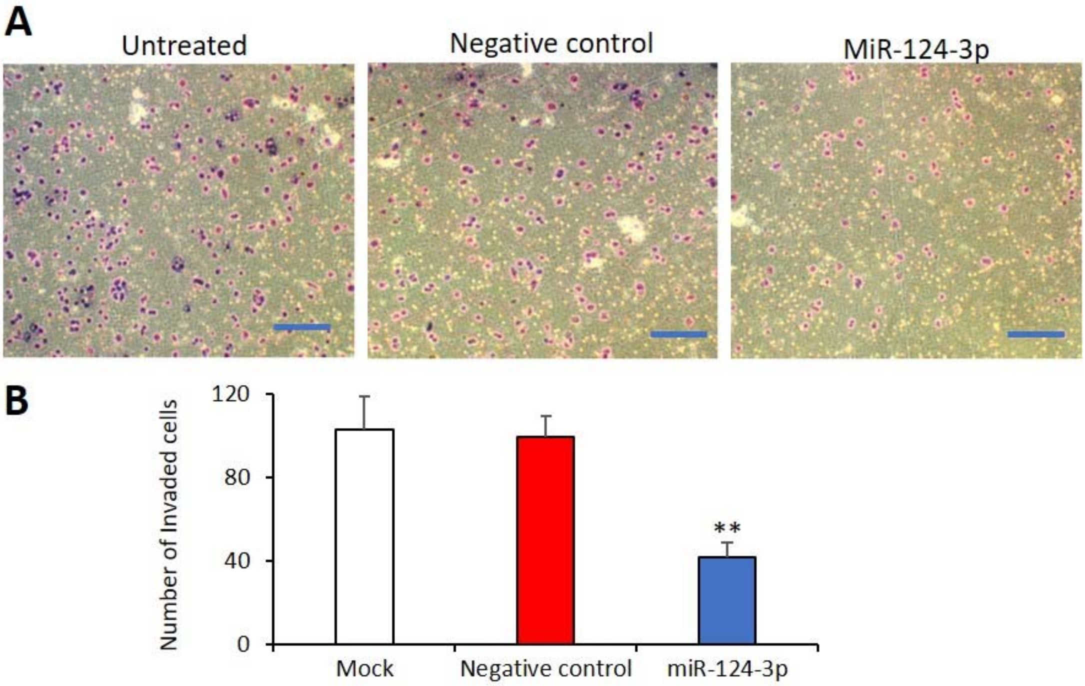

Figure S5 MiR-124-3p inhibits the invasion of MDA-MB-231 cells *in vitro*. (A)

Untreated (Mock) MDA-MB-231 cells or the same cells transfected with negative control or miR-124-3p mimics oligonucleotides were subjected to the Matrigel invasion assay as described in Supplementary Materials and Methods. Magnification bar = 200 µm. (B) The numbers of invaded cells were counted. "** P<0.001" (one-way ANOVA with a Tukey post-hoc test) indicates a significant difference from the negative control.

Figure S6-Primary 4T1 tumors

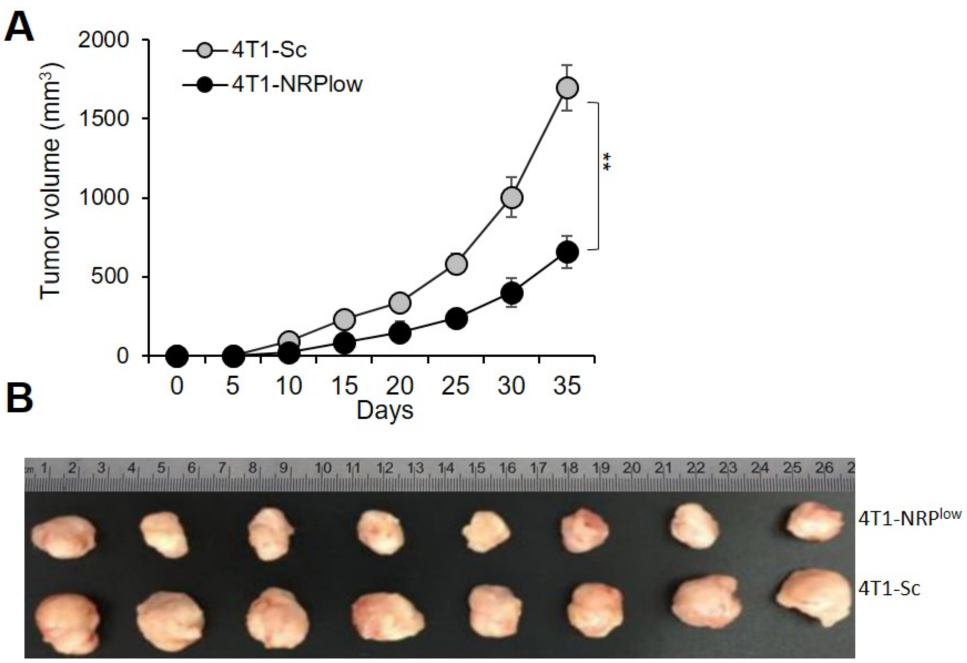

Figure S6. 4T1-Sc and 4T1-NRP^{low} cells were injected into the left inguinal mammary fat pad of BALB/c mice. (A) The size of tumors was measured at indicated time points. (B) Tumors were resected from mice and photographed 35 days later.

Figure S7

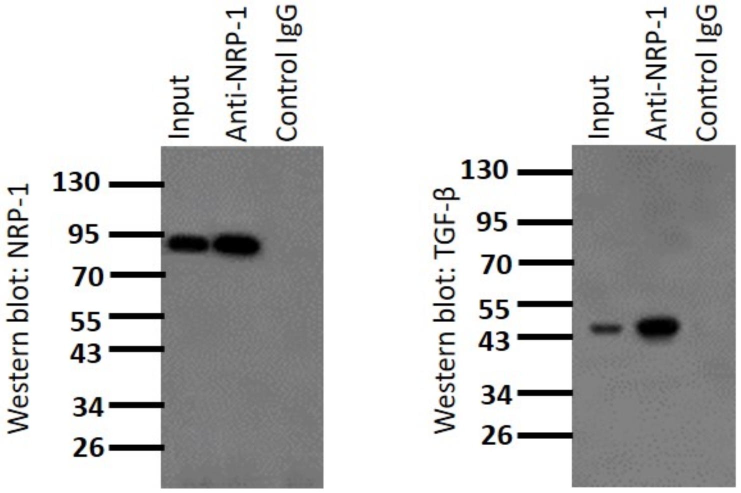

Figure S7 TGF-β binds to NRP-1 in MDA-MB-231 cells. MDA-MB-231 cells incubated with recombinant TGF-β protein (5 ng/ml) for 4 h were lysed. Cell lysates were incubated with protein A-Agarose bound with rabbit anti-NRP-1 Ab or control rabbit IgG. Bound proteins were recovered by Western blot analysis with specific Abs against NRP-1 or TGF-β, respectively. Molecular weight markers are indicated.

Entire images of Western blots in Figures 1, 2, 4, 5, 6, 8 and 9

(The blots in each rectangular box were cropped and shown in the figures)

Figure 1A: Anti-NRP-1

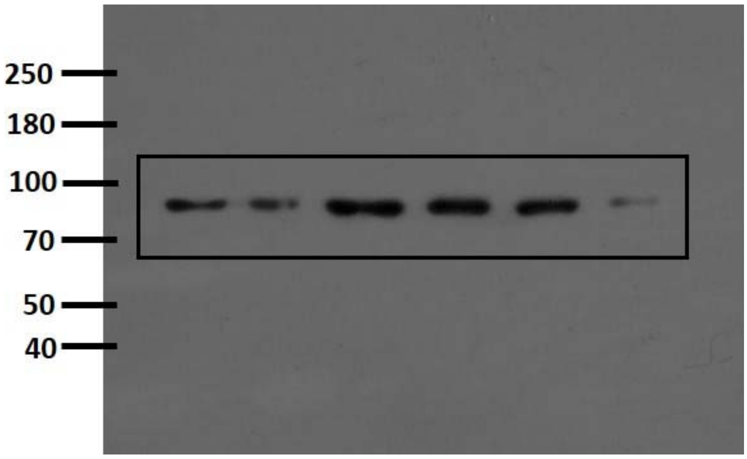

Figure 1A: Anti-β-actin

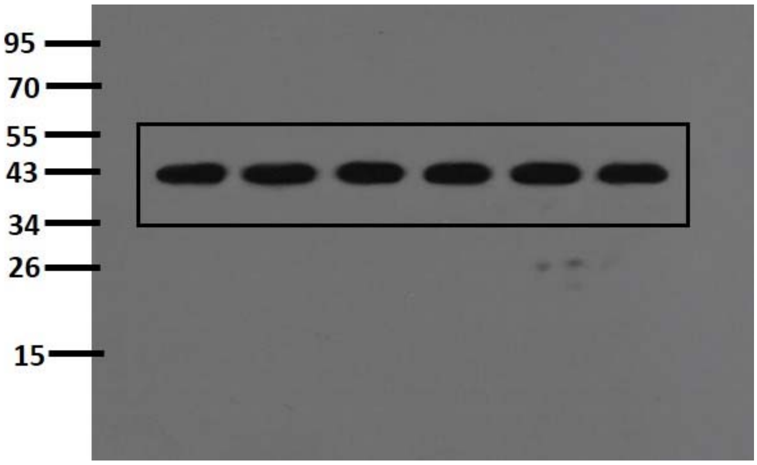

Figure 1D: Anti-NRP-1

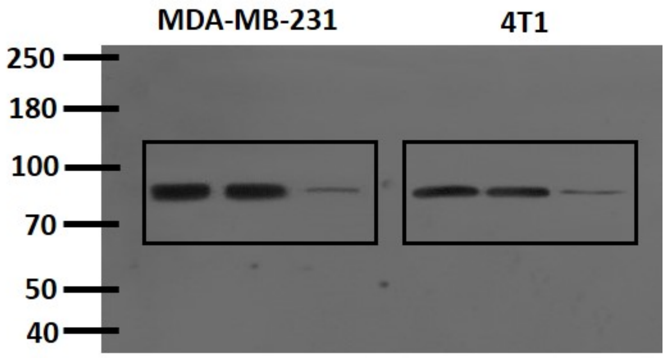

Figure 1D: Anti-β-actin

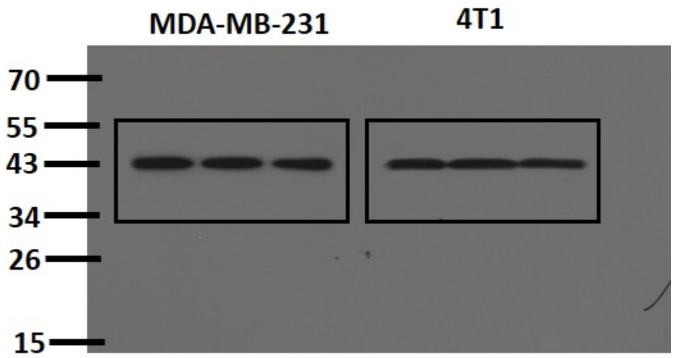

Figure 2B: Anti-NRP-1

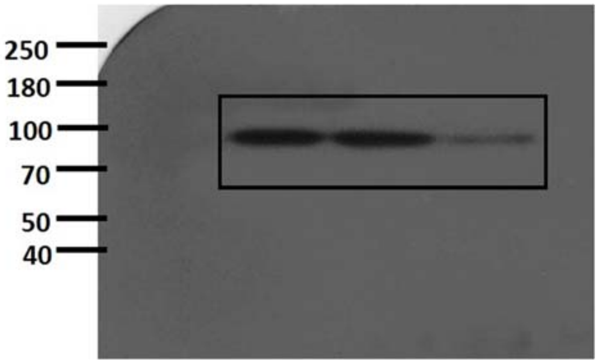

Figure 2B: Anti-cyclin E

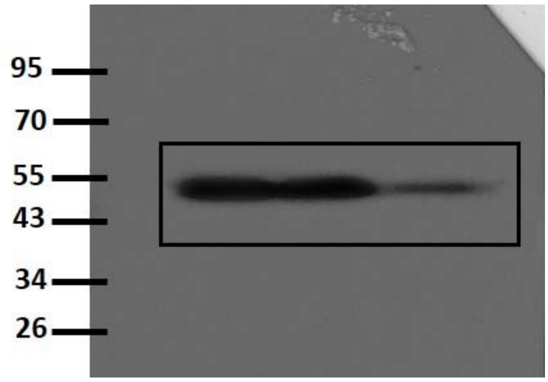

Figure 2B: Anti-Cyclin D1

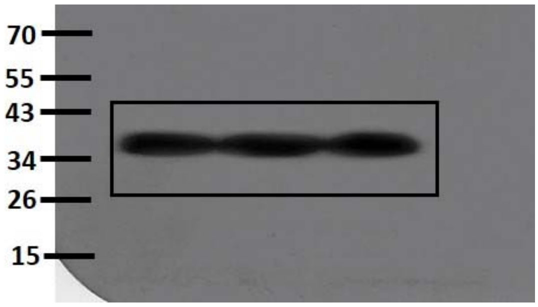

Figure 2B: Anti-CDK2

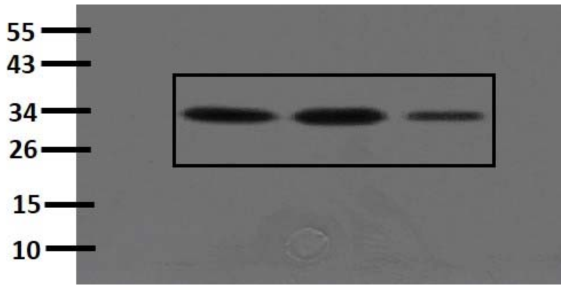

Figure 2B: Anti-p27

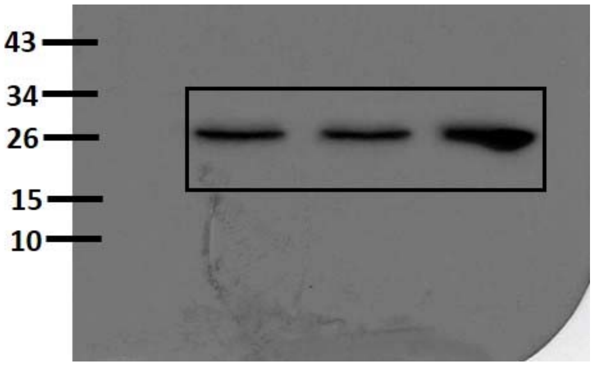

Figure 2B: Anti-p21

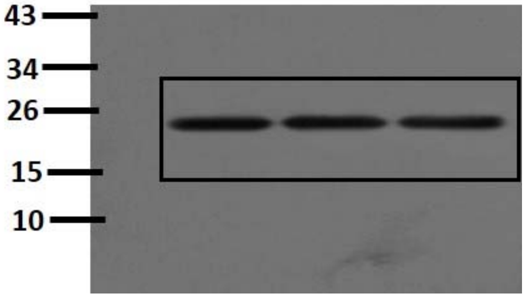

Figure 2B: Anti-β-actin

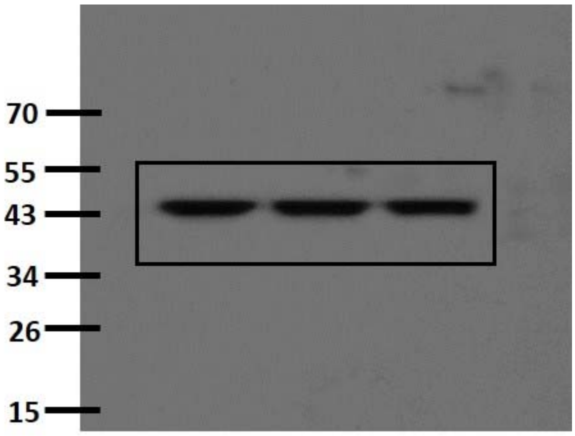

Figure 4C: Anti-NRP-1

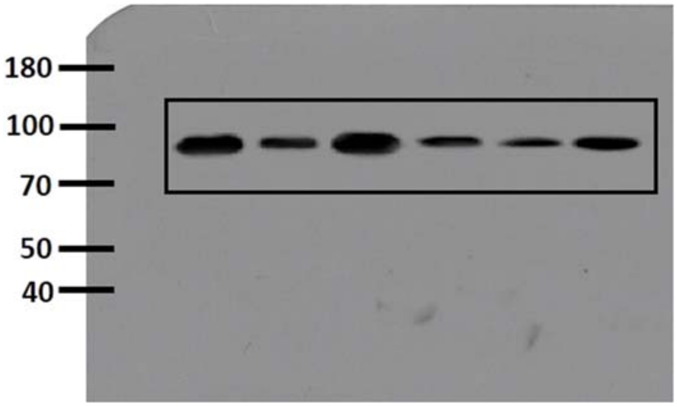

Figure 4C: Anti-β-actin

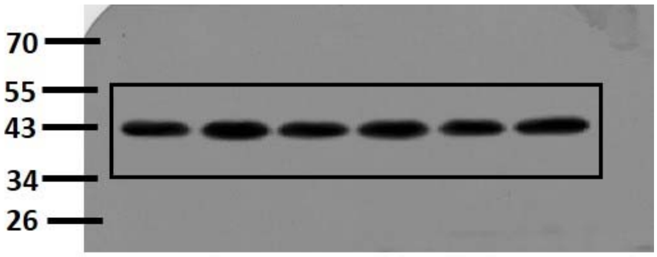

Figure 4D: Anti-NRP-1

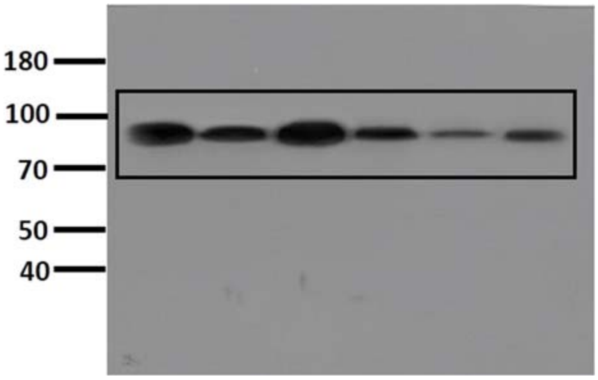

Figure 4D: Anti-β-actin

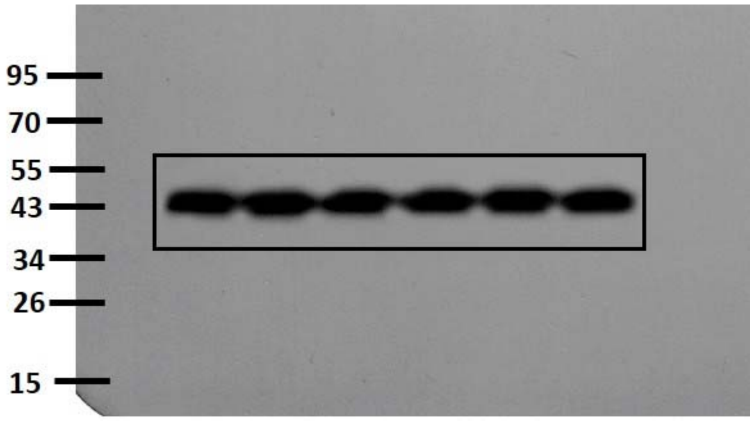

Figure 5B: Anti-NRP-1

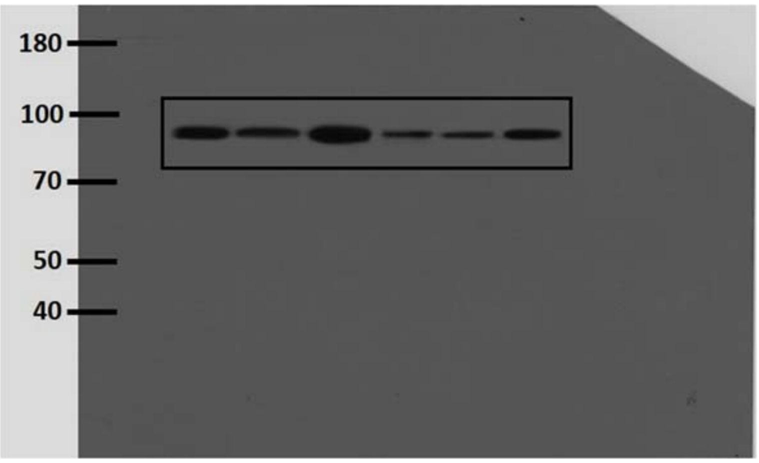

Figure 5B: Anti-cyclin E

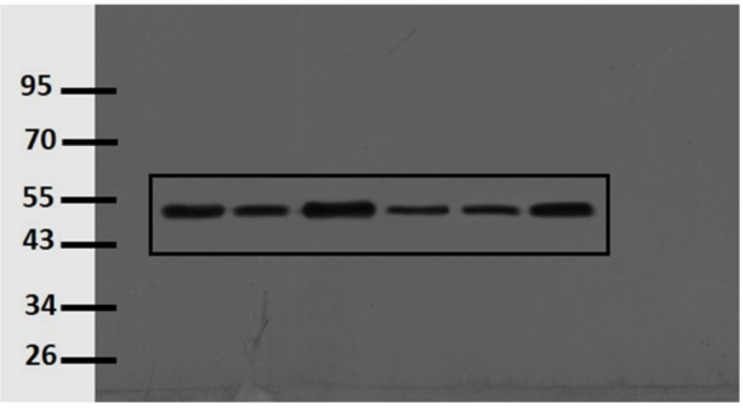

Figure 5B: Anti-CDK2

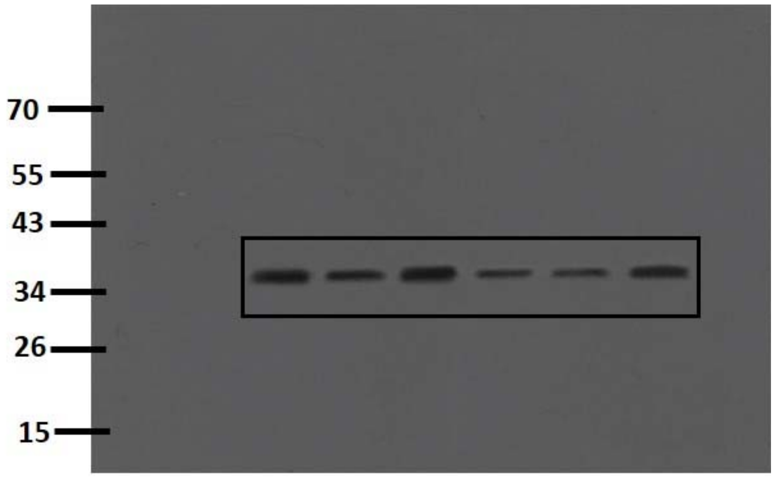

Figure 5B: Anti-p27

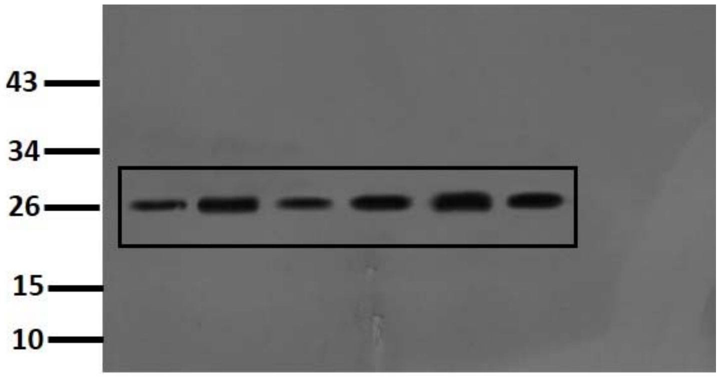

Figure 5B: Anti-β-actin

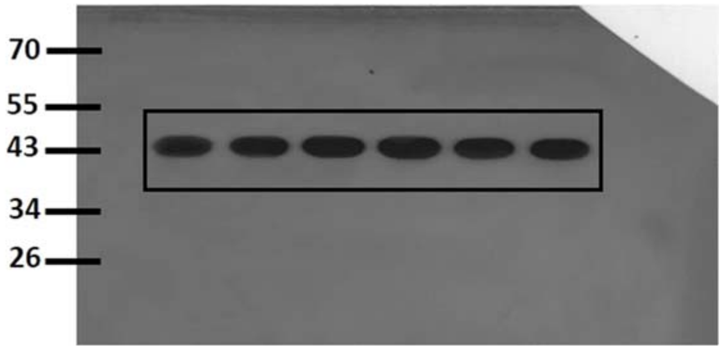

Figure 6E: Anti-NRP-1

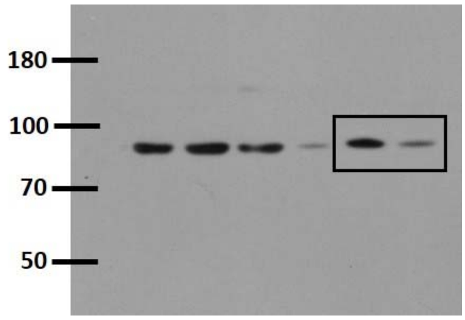

Figure 6E: Anti-E-Cadherin

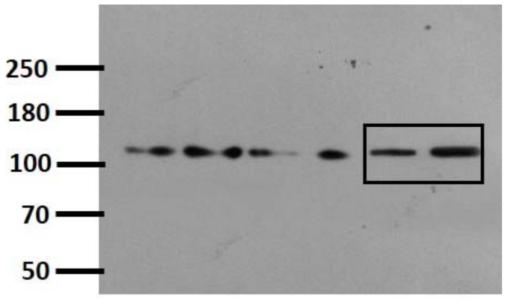

Figure 6E: Anti-N-Cadherin

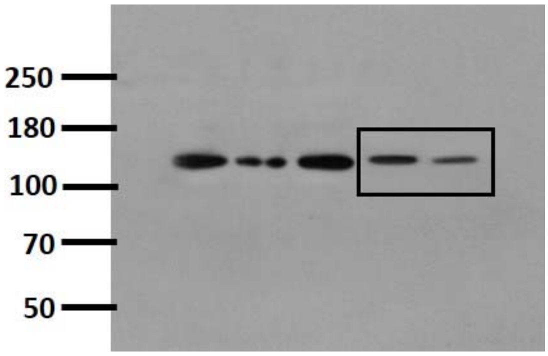

Figure 6E: Anti-MMP2

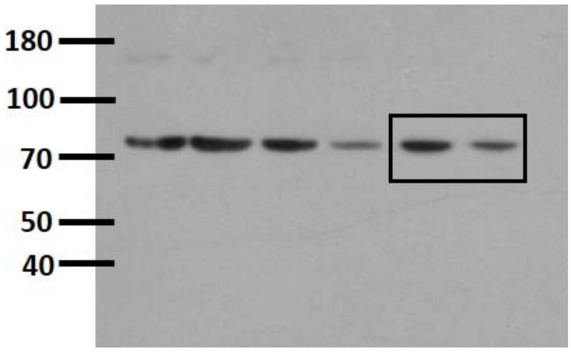

Figure 6E: Anti-MMP9

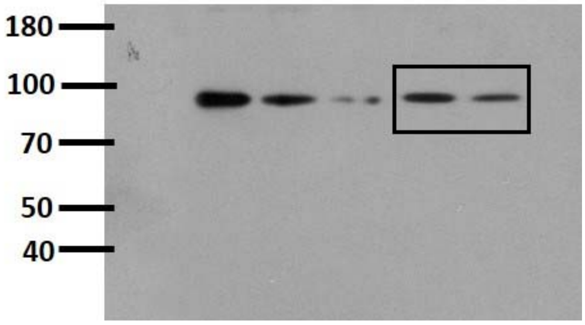

Figure 6E: Anti-β-actin

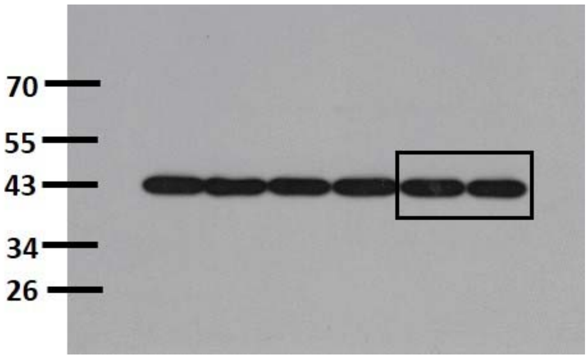

Figure 6H: Anti-E-Cadherin

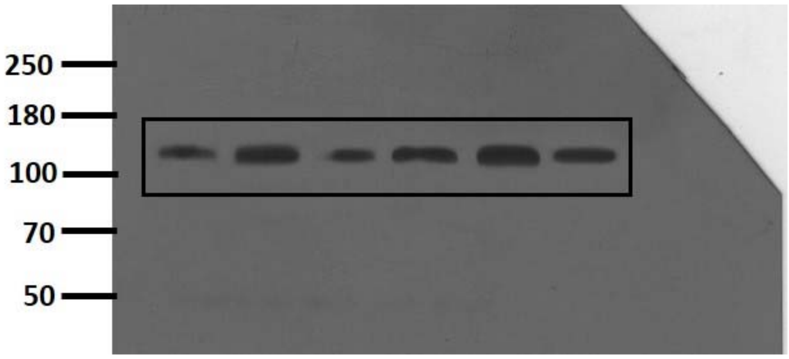

Figure 6H: Anti-N-Cadherin

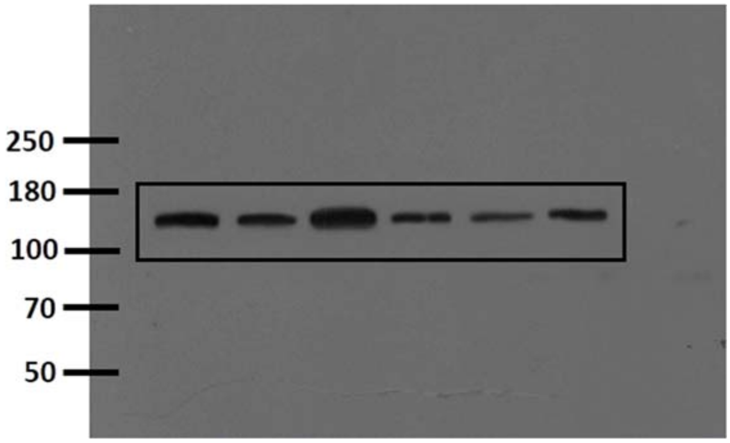

Figure 6H: Anti-β-actin

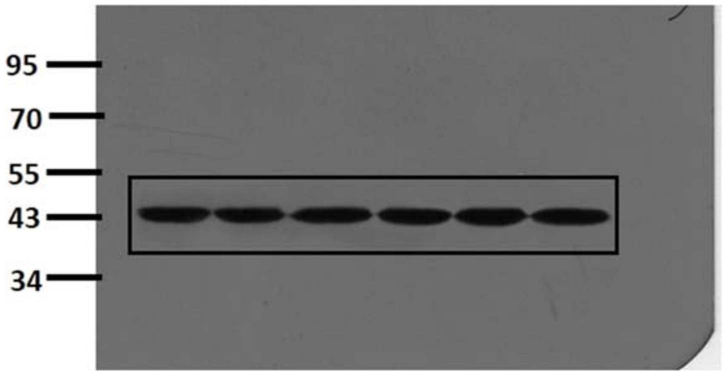

Figure 8C: Anti-NRP-1

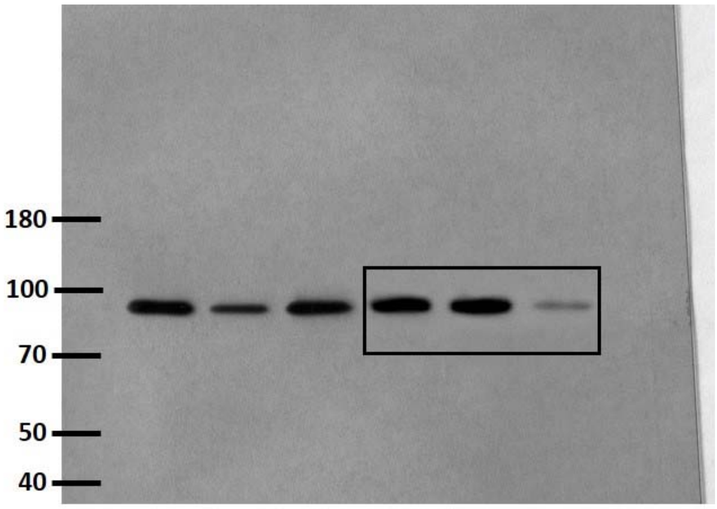

Figure 8C: Anti-cyclin E

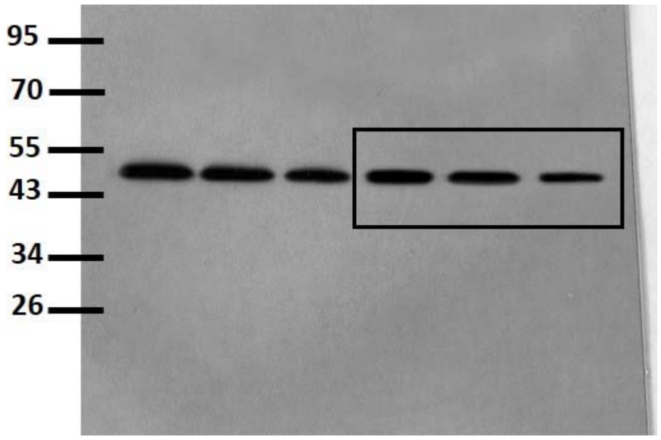

Figure 8C: Anti-CDK2

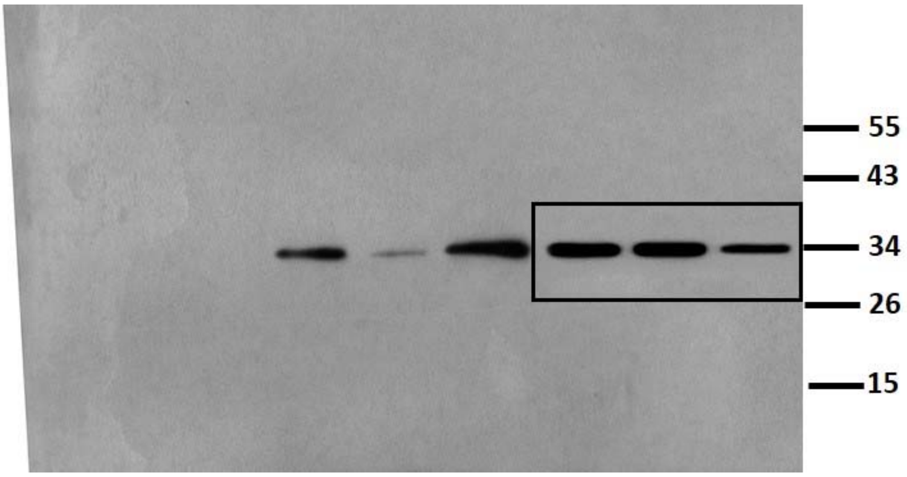

Figure 8C: Anti-p27

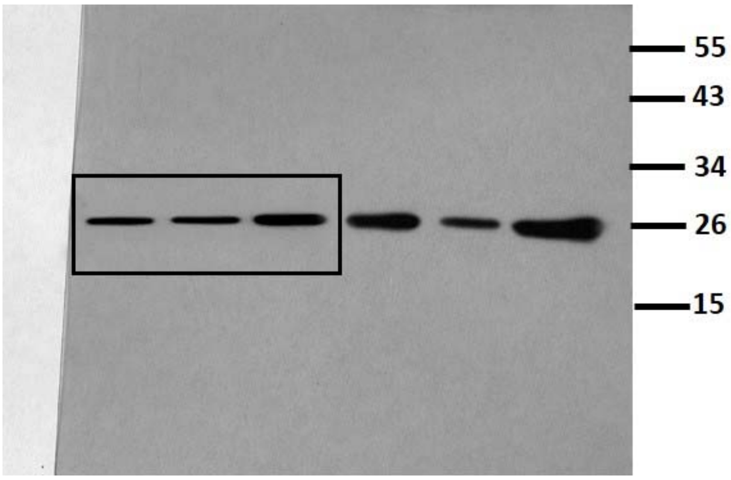

Figure 8C: Anti-E-Cadherin

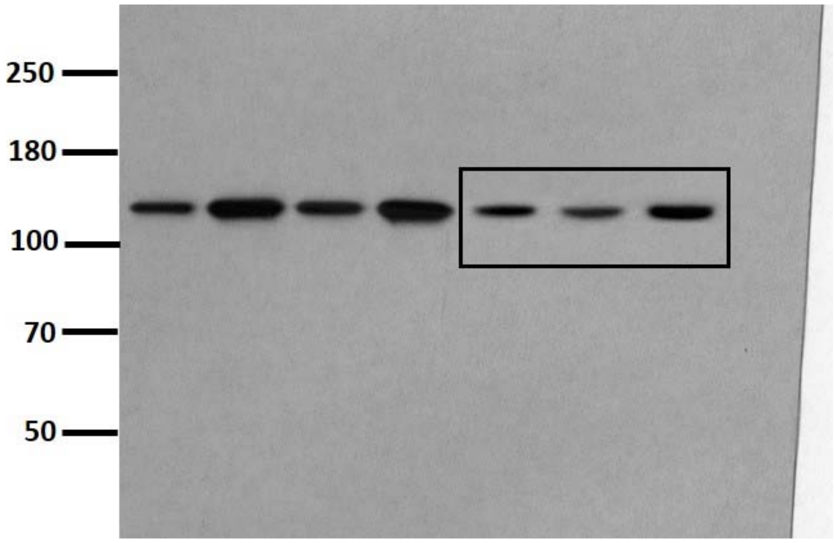

Figure 8C: Anti-N-Cadherin

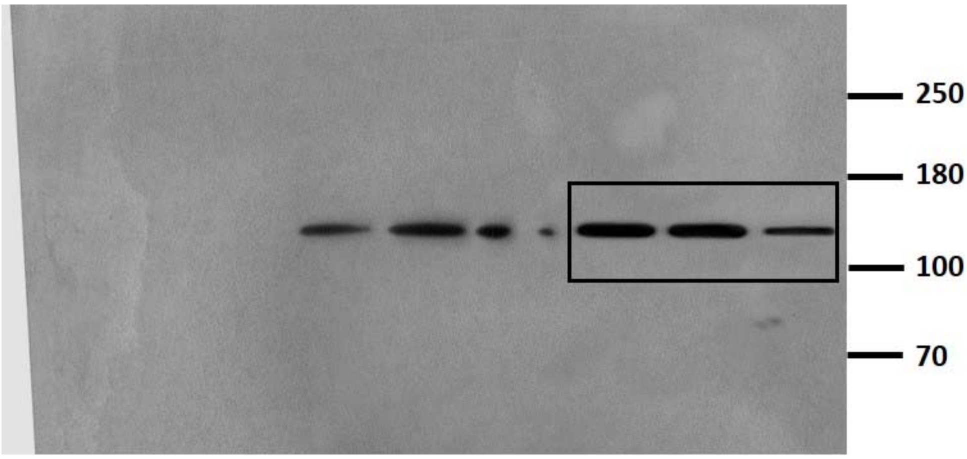

Figure 8C: Anti-β-actin

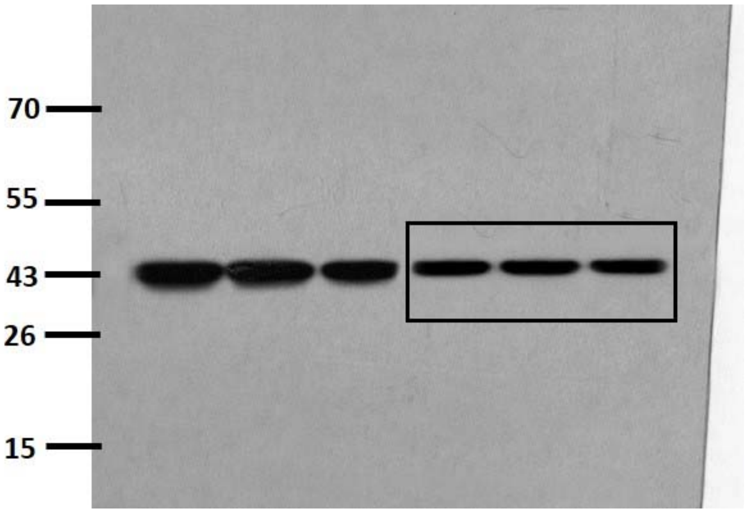

Figure 9A: Anti-NRP-1

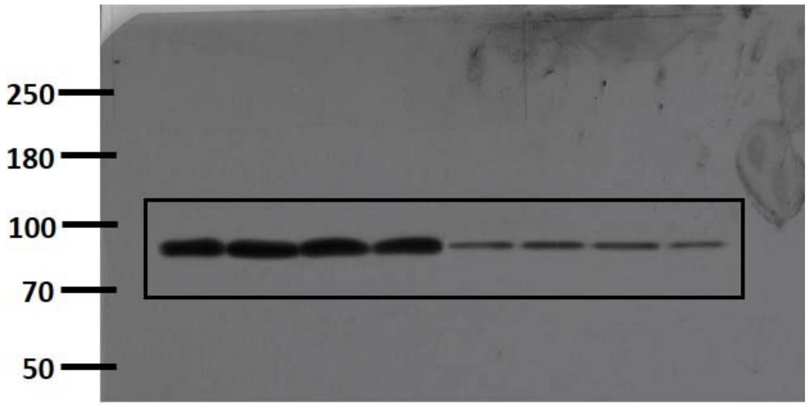

Figure 9A: Anti-TGF-βR I

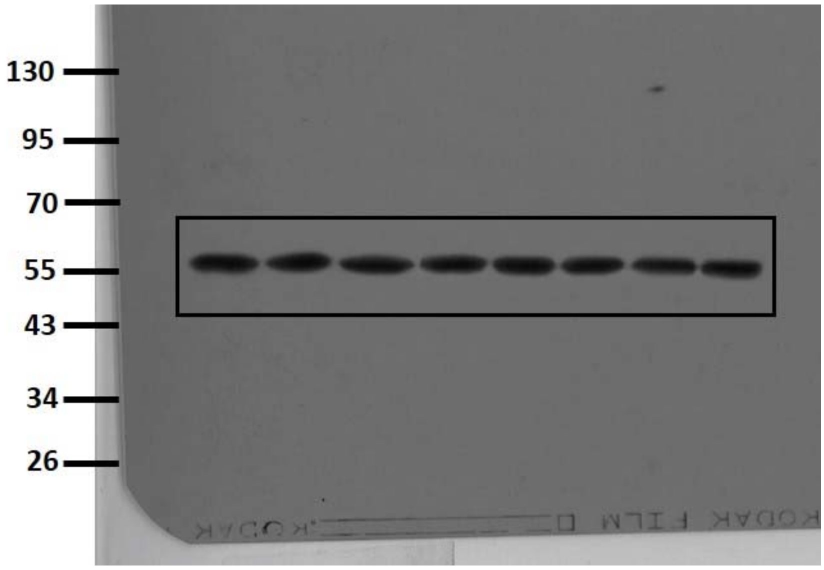

Figure 9A: Anti-Smad2/3

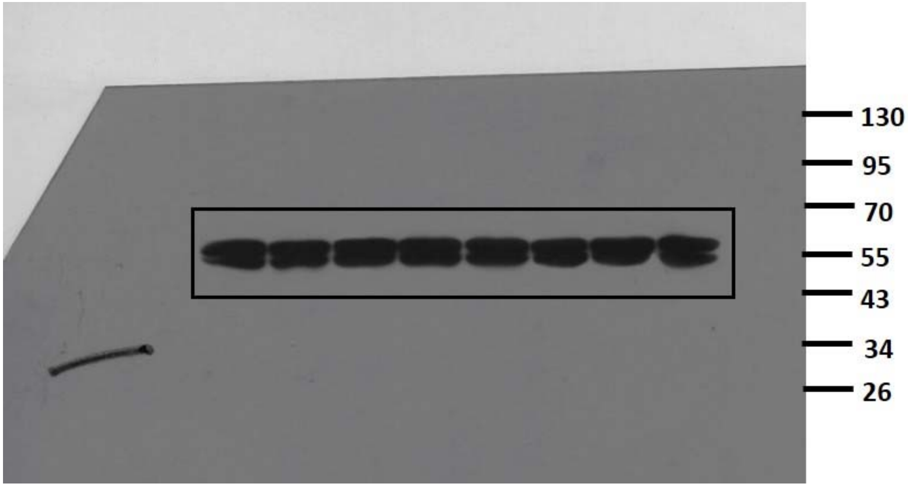

Figure 9A: Anti-Smad4

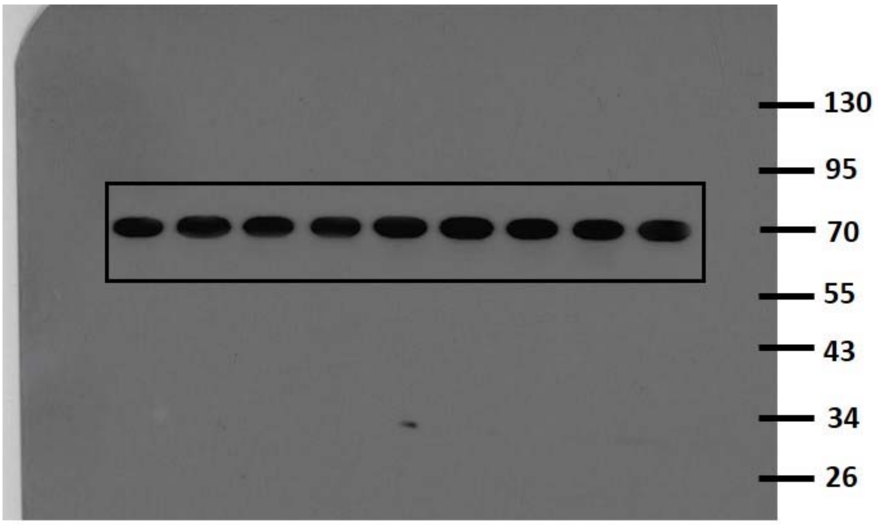

Figure 9A: Anti-E-Cadherin

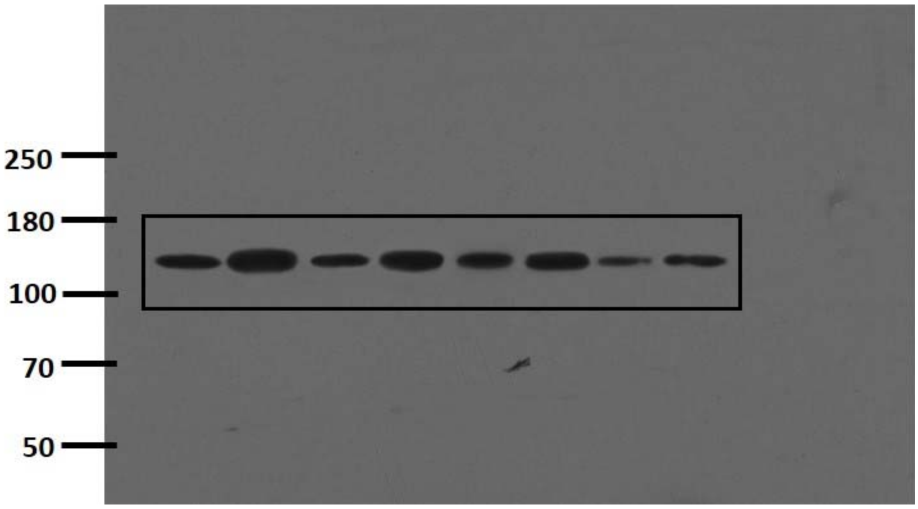

Figure 9A: Anti- β-actin (as an internal control for Western blots for Anti-NRP-1, Anti-TGF-βR I, Anti-Smad2/3, Anti-Smad4 and Anti-E-Cadherin in Figure 9)

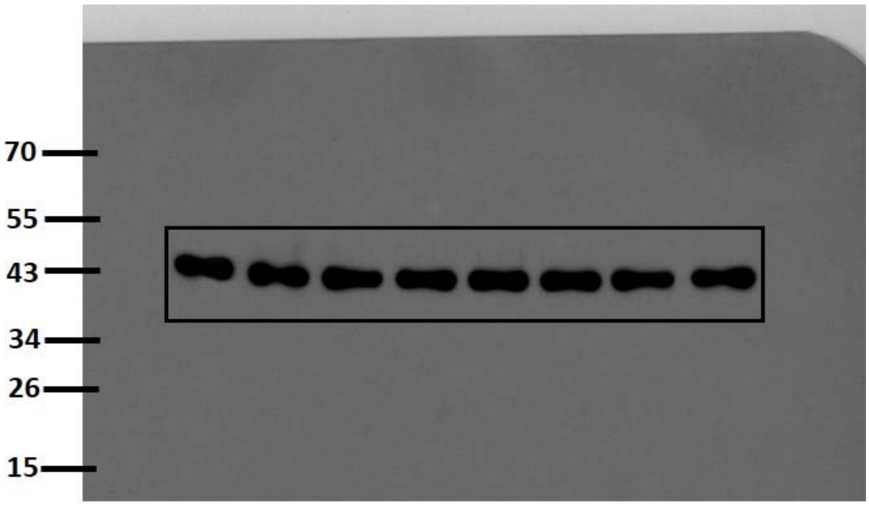

Figure 9A: Anti-p-TGF-βR I

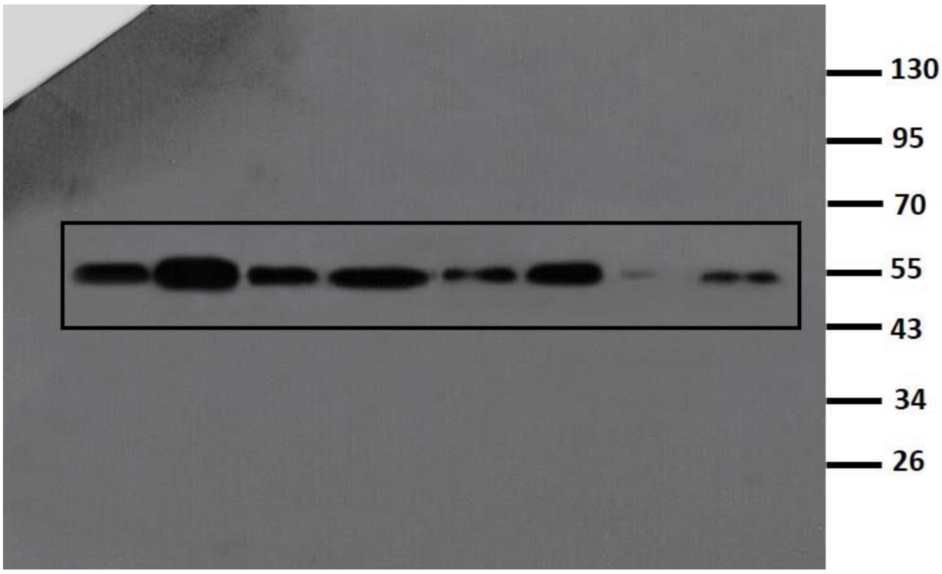

Figure 9A: Anti-p-Smad2/3

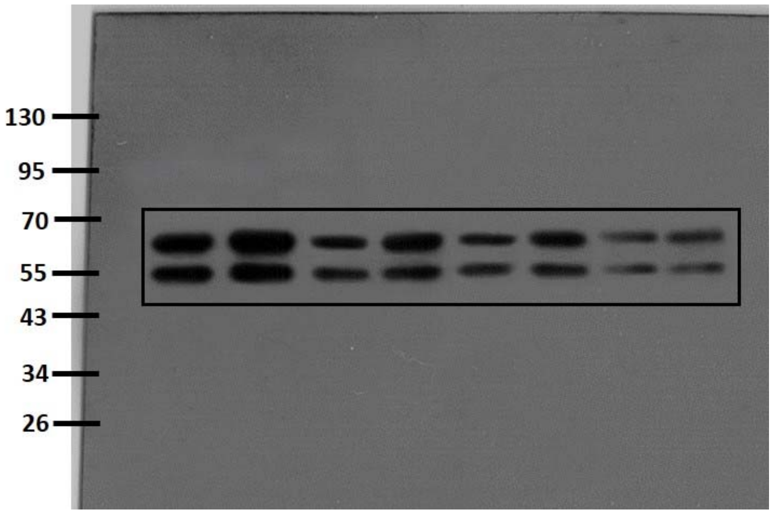

Figure 9A: Anti-Snail

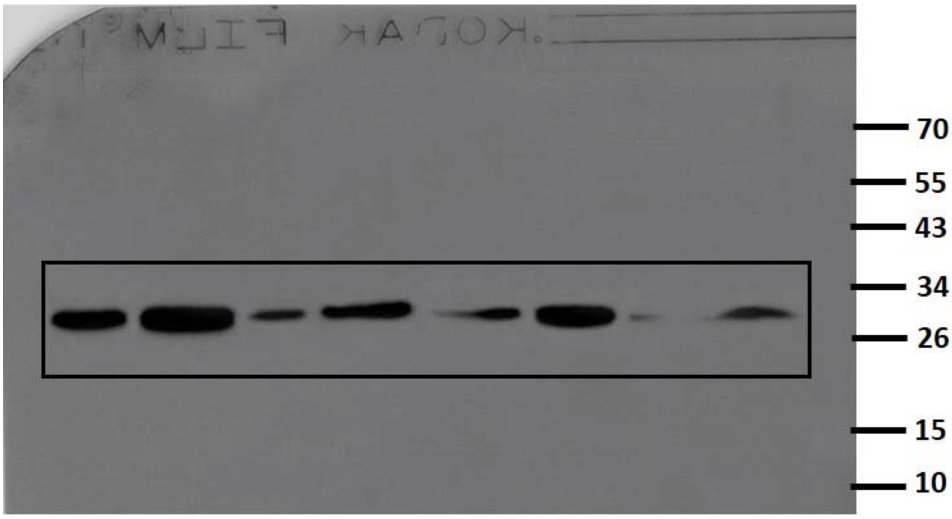

Figure 9A: Anti-N-Cadherin

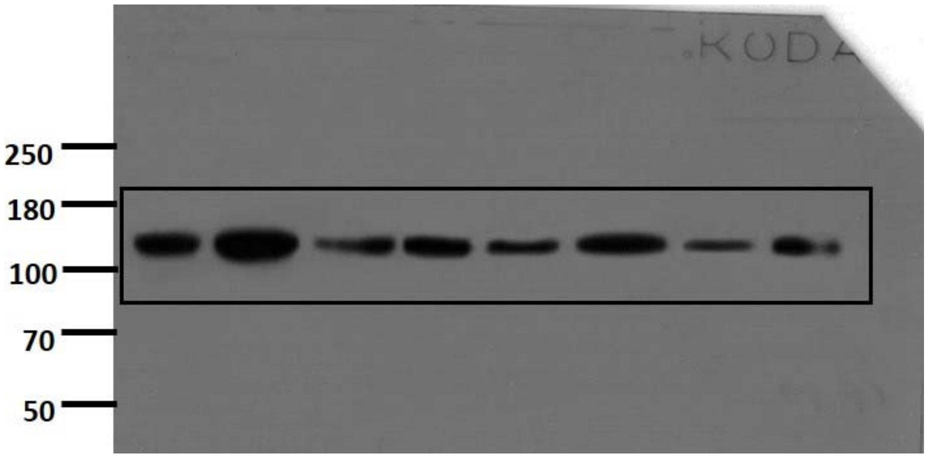

Figure 9A: Anti- β-actin (as an internal control for Western blots for Anti-p-TGF-βR I, Anti-p-Smad2/3, Anti-Snail and Anti-N-Cadherin in Figure 9)

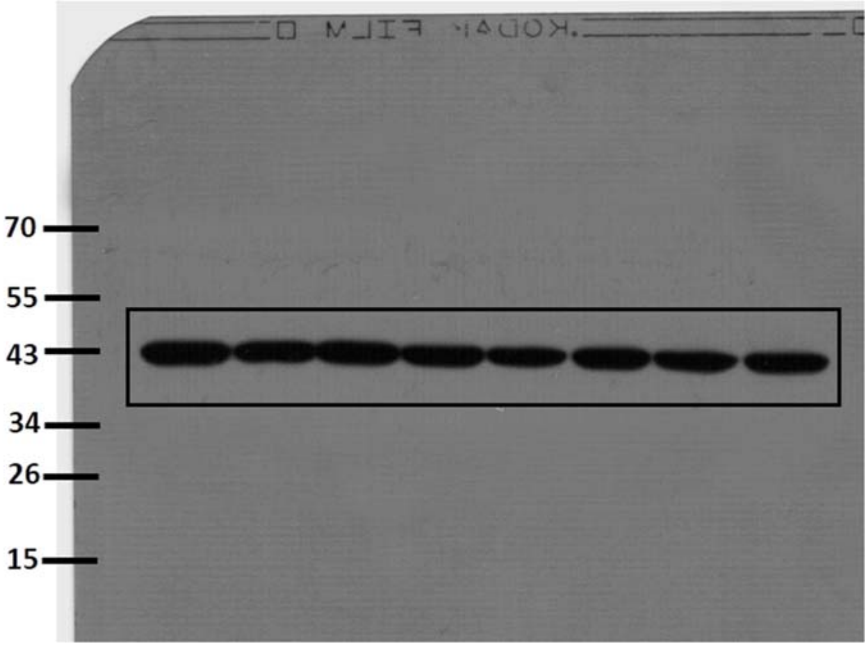

Supplement: Supplementary file 1 [file DataSheet_1.pdf]
